# Flexible pyroelectric energy harvesters from nanocomposites of liquid crystal elastomers/lead zirconate titanate nanoparticles

**DOI:** 10.1126/sciadv.adt6136

**Published:** 2025-02-12

**Authors:** Shangsong Li, Yuchen Wang, Zixiao Liu, Baohong Chen, Mingzhu Liu, Ximin He, Shu Yang

**Affiliations:** ^1^ Department of Materials Science and Engineering, University of Pennsylvania, 3231 Walnut Street, Philadelphia, PA 19104, USA.; ^2^ Department of Materials Science and Engineering, University of California, Los Angeles, 410 Westwood Plaza, Los Angeles, CA 90095, USA.

## Abstract

Pyroelectric materials that can generate electric charges when subjected to temperature changes are of interest for renewable energy. However, current flexible pyroelectric energy harvesters suffer from low output. Here, we present a nanocomposite of liquid crystalline elastomer (LCE) and pyroelectric lead zirconate titanate (PZT) nanoparticles and demonstrate a flexible heat harvesting device with high output. The overall pyroelectricity is enhanced by the secondary pyroelectricity generated from the thermal stress imposed on the LCE. Calculations and simulations corroborate with experiments, suggesting that the monodomain LCE/PZT with fixed boundaries offers the most enhancement. At a maximum heating rate of 0.20 kelvin per second, the fixed monodomain film (42.7 weight % PZT) shows an output current of 2.81 nanoamperes and a voltage of 6.23 volts, corresponding to a pyroelectric coefficient *p* of −4.01 nanocoulombs per square centimeter per kelvin, 49% higher than that of the widely used polyvinylidene fluoride. Our energy harvester can charge capacitors and power electronic devices such as light-emitting diodes.

## Teaser

LCE/PZT composites demonstrate a flexible heat harvesting device with enhanced pyroelectric output caused by LCE thermal stress.

## INTRODUCTION

The escalating global energy demands call for the development of sustainable and environmentally friendly renewable energy. Heat, a ubiquitous form of energy, represents a major resource. However, more than 60% of the total energy generated across all sources in the US is dissipated as wasted heat (*1*). Harnessing the wasted heat not only promises a reduction in carbon emissions but also opens the door to sustainable energy. The most direct method of converting heat to electricity relies on the Seebeck effect, which uses the temperature gradient across a thermoelectric device to drive charge carriers from the hot side to the cold side (*2*). However, in an outdoor environment, the temperature distribution is rather uniform, rendering the conventional Seebeck effect less effective for energy harvesting.

Compared to the Seebeck effect, the pyroelectric effect offers an attractive alternative solution. It harvests thermal energy from time-dependent temperature fluctuation, a phenomenon commonly encountered in daily life. The pyroelectric effect arises when pyroelectric materials experience a change in the polarization level with temperature change, described quantitatively by the pyroelectric coefficient (*3*, *4*), which is composed of two parts. One is the primary pyroelectric effect, namely, a change in temperature induces a change in electric displacement directly at a constant strain. The other is the secondary pyroelectric effect, namely, a temperature change induces stress in the material by thermal expansion, and then, the stress induces additional electric displacement via a piezoelectric process (*5*). The secondary pyroelectric effect is a piezoelectric effect in essence, while the stress stimuli are not applied directly but from the change of initial temperature (*6*).

Pyroelectric nanogenerators have been developed to harvest waste heat on the basis of different pyroelectric materials such as zinc oxide (ZnO) (*7*), barium titanate (BaTiO_3_) (*8*), lead zirconate titanate (PZT) (*9*), potassium niobate (KNbO_3_) (*10*), and polyvinylidene fluoride (PVDF) (*11*–*13*). While ceramic materials offer a high magnitude of pyroelectric coefficient, *p* (up to −80 nC cm^−2^ K^−1^) (*9*), their high rigidity greatly limits their applications to flexible, wireless, and wearable electronics and smart sensors. Polymers such as PVDF are commonly used in flexible pyroelectrics. However, their pyroelectric performance is limited, e.g., *p* = −2.70 nC cm^−2^ K^−1^ for PVDF (*10*, *13*). Therefore, researchers have explored the fabrication of hybrid generators by introducing piezoelectric effects to harness both pyroelectricity and piezoelectricity from a single nanogenerator by applying heat and compression simultaneously, since all pyroelectric materials also exhibit piezoelectric properties (*14*, *15*). However, the pyroelectric signal itself is not enhanced in these material systems; rather, the enhancement comes from responses from other types of stimuli. That is, two or more different stimuli need to be applied simultaneously, which is inconvenient in practice. It will be highly desired to enhance the overall pyroelectricity by intensifying the secondary pyroelectric effect from heat change only. The secondary pyroelectric coefficient is an intrinsic property of the pyroelectric materials. McKinley and Pilon (*16*) propose that thermal expansion because of phase change can increase the pyroelectric energy conversion density from 12.5 to 28.9 kJ m^−3^ by using 0.72PbMg_1/3_Nb_2/3_O_3_-0.28PbTiO_3_ (PMN-28PT) at a frequency higher than 0.0211 Hz. Li *et al.* (*17*) show that the pyroelectricity coefficient of the ferroelectric BaTiO_3_ can be increased 4.8 times when operating near its Curie temperature (130°C) because of the stress contribution during phase change. However, operating near the Curie temperature also leads to a faster depolarization of the ferroelectric materials toward failure of the pyroelectric devices.

Liquid crystalline elastomers (LCEs) with the intrinsic molecular anisotropy of the liquid crystal (LC) mesogens can generate stress and undergo shape transformations upon heating above the nematic-to-isotropic phase transition temperature, *T*_NI_ (*18*). Below *T*_NI_, LC molecules can be aligned all in one direction, forming a so-called monodomain. When LC molecules are aligned only in a small domain with an approximate size of 2.5 μm (*19*), polydomains are formed with each domain randomly oriented against each other. Upon heating above *T*_NI_, LC molecules lose their orientations and become isotropic. For monodomain LCEs, the sample shrinks from the aligned directions, exerting large stress. For polydomain LCEs, the stress generated in individual domains is canceled out in a macroscopic sample.

By introducing functional nanoparticles (NPs), LCE nanocomposites (*18*) can be made responsive to light (*20*), electric field (*21*), and magnetic field (*22*) for applications including artificial muscles (*21*), soft robotics (*20*), magnetic memory (*23*), and cell culture (*24*). Nevertheless, their applications in energy harvesting have received comparatively less attention (*25*, *26*). Given the large thermal stress of LCEs, they are promising candidates for integration with pyroelectric materials such that the thermally induced stress in LCEs could be transferred to pyroelectric materials to generate a secondary pyroelectric effect that enhances the original pyroelectric signal. Wei *et al.* (*27*) apply a layer of LCE on the poly(dimethylsiloxane) (PDMS) side of the PVDF/PDMS triboelectric device, where the bending of the LCE layer upon heating generates triboelectric and secondary pyroelectric signals. Han *et al.* (*28*) fabricate a PVDF/LCE bilayer film, and film bending facilitates the simultaneous capture of primary and secondary pyroelectric signals. However, their overall performance remains low with the maximum output voltage of 4.5 mV, since the actuation stress generated in the LCE layer cannot be effectively transferred to PVDF in these layered systems.

Herein, we design and fabricate the LCE composite embedded with PZT NPs, achieving an output current of 2.81 nA and a voltage of 6.23 V, corresponding to *p* = −4.01 nC cm^−2^ K^−1^, which is higher than literature values for flexible pyroelectric materials (see table S1). Since the alignment (monodomain versus polydomain) and boundary conditions of the LCE film (two ends fixed or nonfixed) can affect the effective interactions between the LCE and PZT NPs, we investigate their influence on the output performance through experiments, theoretical calculations, and simulations. The LCE/PZT composite can power light-emitting diodes (LEDs) with multiple cycles, demonstrating the potential applications of flexible energy harvesters and self-powered devices.

## RESULTS

### Materials fabrication

**Fig. 1.**
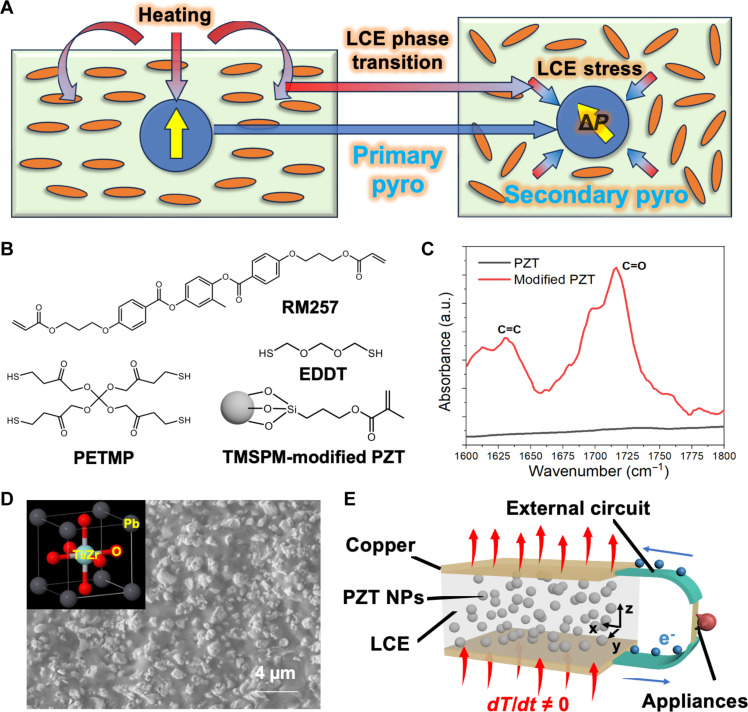
Concept and materials for preparation of the PZT/LCE composite. (**A**) Schematic of the core concept using the secondary pyroelectric effect induced by the interactions between LCE thermal stress and PZT particles to enhance the overall pyroelectric effect. (**B**) Molecular structures of the monomers, chain extenders, and cross-linkers to prepare LCE and the surface-functionalized PZT. (**C**) FTIR spectrum of the pristine and modified PZT NPs. a.u., arbitrary units. (**D**) Cross-sectional SEM image of the LCE/PZT film with a PZT NP loading of 42.7 wt %. Inset: crystal structure of PZT. (**E**) Schematic of the LCE/PZT energy harvester.

In the LCE/PZT composite, we expect PZT NPs to generate the primary pyroelectricity, while the thermal stress generated from the LCE matrix could be transferred to PZT NPs to enhance the secondary pyroelectricity (Fig. 1A). The LCE film is prepared by the two-step thiol-acrylate Michael addition reactions (*29*). First, the LC monomer 1,4-bis-[4-(3-acryloyloxypropyloxy)benzoyloxy]-2-methylbenzene (RM257), the chain extender 2,2′-(ethylenedioxy) diethanethiol (EDDT), and the cross-linker pentaerythritol tetrakis(3-mercaptopropionate) (PETMP) (Fig. 1B) are mixed together with PZT NPs that are surface modified with methacrylate end groups. A 1.05:1 molar ratio of acrylates to thiols and a 13:1 molar ratio of EDDT to PETMP are applied to enhance the flexibility. Then, the catalyst, dipropylamine (DPA), is added to the mixture, followed by casting it into a film to complete the thermal curing at 90°C to prepare the polydomain samples (25 mm by 10 mm by 1 mm). The monodomain samples (50 mm by 7 mm by 0.7 mm) are prepared by stretching the as-cast films to 100% strain before thermal curing (see details in Materials and Methods). The monodomain alignment of the LCE films is verified by the polarized optical microscope equipped with cross-polarizers, where the brightness of the polarized optical microscopy images changes from dark to bright to dark when rotating the film by 90° (fig. S1). After mixing with PZT NPs, they can still show reversible contraction/elongation upon heating/cooling (fig. S2).

The resulting Young’s moduli of the polydomain and monodomain LCE/PZT (27.1 wt % PZT) are 1.0 and 1.7 MPa in the longitudinal direction, respectively (fig. S3), much smaller than that of PVDF, 1.6 to 2.8 GPa (*30*–*32*). In most LCE composites for actuation applications, nanofiller loadings are typically less than 4 wt %, above which the viscosity of the mixture is increased substantially, and thus, the nanofillers cannot disperse uniformly (*33*–*35*). To address this issue, we surface modify PZT NPs with 3-(trimethoxysilyl)propyl methacrylate (TMSPMA), which can covalently cross-link with the LCE matrix via the thiol groups (*36*). As a result, a very high loading (10 to 60 wt %) of PZT NPs can be introduced in our LCE matrix, which is important to generate larger electric signals. Compared to the Fourier transform infrared (FTIR) spectrum of the pristine PZT NPs, the appearance of the carbonyl and alkene groups at 1710 and 1630 cm^−1^, respectively, in the FTIR spectrum of the modified PZT NPs clearly indicates successful surface functionalization (Fig. 1C). From the scanning electron microscopy (SEM) images of PZT NPs and the cross-sectional view of the LCE/PZT films (Fig. 1D and fig. S4), PZT NPs (average diameter, 240 nm) are randomly distributed in the LCE matrix without any notable aggregation. Besides high loading, the covalent bonding between PZT NPs and the LCE matrix also enhances their interfacial contact, presumably facilitating the efficient transfer of thermal stress from LCE to PZT NPs (*37*), which is critically important to generate a substantial secondary pyroelectric effect. We use thermal curing here to ensure uniform cross-link in a rather thick film (1 mm).

The cross-linked LCE/PZT films are sandwiched between two copper films as the top and bottom electrodes (Fig. 1D) for poling at an electric field of 1 kV mm^−1^ for 4 hours, aligning the dipole moments of PZT NPs across the sample thickness. When the temperature changes, the primary pyroelectric effect of PZT, combined with the secondary pyroelectric effect from the thermally induced stress in LCE, is expected to provide power to an external circuit (Fig. 1D). In this way, the energy from temperature fluctuations in the surroundings can be harvested.

### Pyroelectric output of the polydomain LCE/PZT

**Fig. 2.**
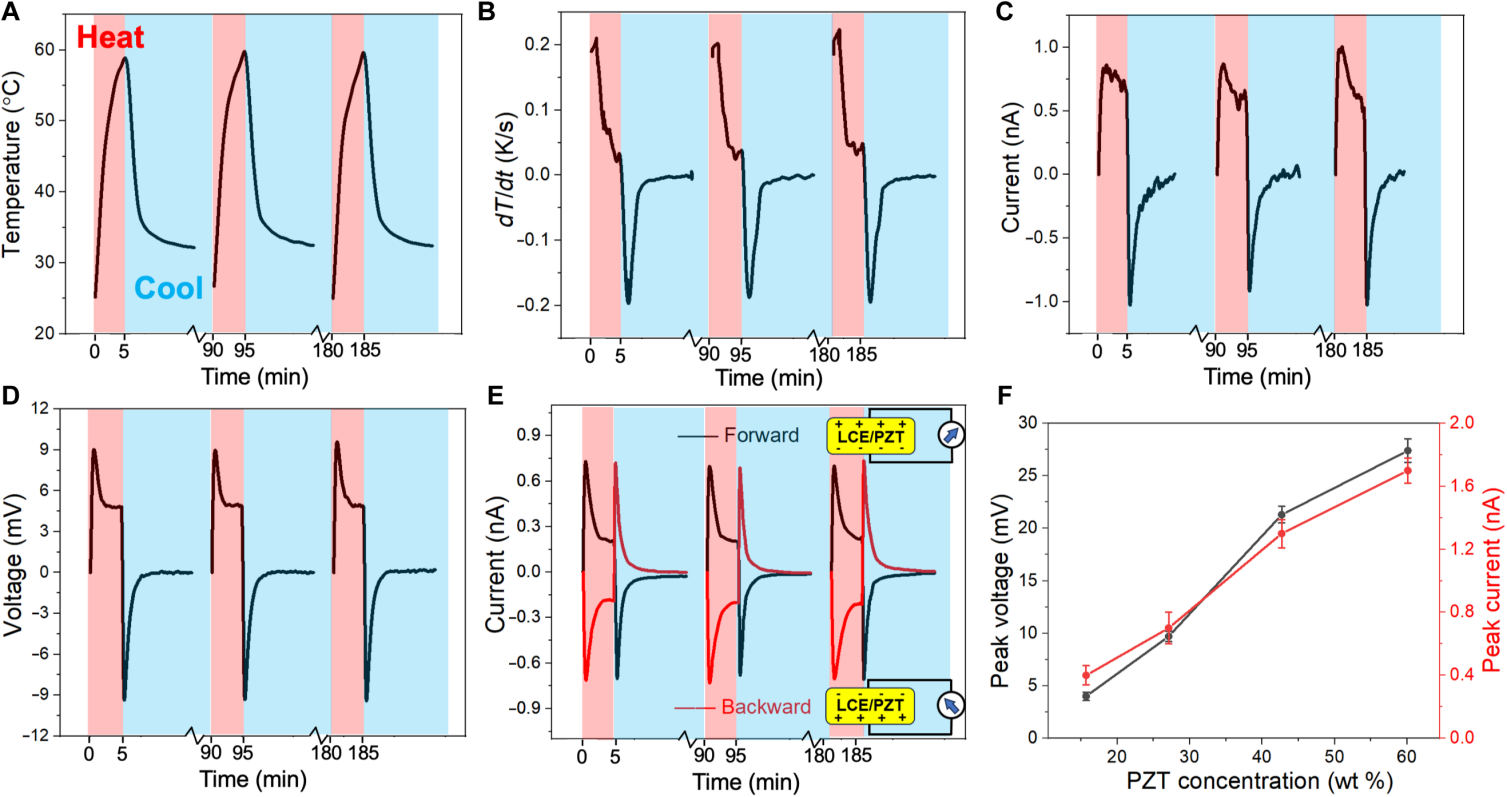
Pyroelectric performance of the polydomain LCE/PZT composite film (25 mm by 10 mm by 1 mm). In (A) to (E), pink color bars stand for the heating process (5 min), and blue color bars stand for the cooling process (85 min). (**A**) Cyclic temperature profiles with temperatures varied from 25° to 58°C, with the cooling part truncated. (**B**) Derivative of the cyclic temperature profiles to time. (**C** and **D**) Short-circuit current (C) and open-circuit voltage (D) profiles of the polydomain LCE/PZT film with 27.1 wt % PZT NPs. (**E**) Short-circuit current profiles of the polydomain LCE/PZT film with 27.1 wt % PZT NPs at both forward and backward connections. (**F**) Peak voltage and peak current of the polydomain LCE/PZT films with different PZT concentrations: 15.7, 27.1, 42.7, and 60.1 wt %.

The output current and voltage of the polydomain LCE/PZT composite are first investigated to understand the basic behaviors of the LCE/PZT system. The LCE/PZT film is irradiated by a torch lamp with repeated heating (for 5 min) and cooling (for 85 min) cycles between 25° and 58°C, as shown in Fig. 2A. The cooling time is relatively long to make sure that the temperature returns to 25°C after one cycle (see the whole profile in fig. S5). It is noted that the peak temperature of 58°C is above the *T*_NI_ (52.5°C) of the LCE/PZT composite (fig. S6). For pyroelectricity, the output current *I* can be described in (*38*)

$$I=\mathrm{pA}\frac{\mathrm{dT}}{\mathrm{dt}}$$ (1)

where *A* is the effective area of the pyroelectric device, and $\frac{\mathrm{dT}}{\mathrm{dt}}$ is the rate of temperature change. Since *p* and *A* are fixed for a certain system, the output current should have a similar profile as $\frac{\mathrm{dT}}{\mathrm{dt}}$, which is calculated with a maximum heating rate of 0.20 K s^−1^ (Fig. 2B). This is verified in experiments on a polydomain LCE/PZT film with 27.1 wt % PZT NPs (Fig. 2C), which shows a peak output current of 0.76 nA at the maximum heating rate of 0.20 K s^−1^, and the current changes direction when the $\frac{\mathrm{dT}}{\mathrm{dt}}$ changes its sign. The voltage profile is also measured with a peak voltage of 9.3 mV when the internal impedance of the voltmeter is 10 megohms (Fig. 2D). Since the output voltage of the pyroelectric materials is highly dependent on voltmeter parameters, a voltmeter with larger impedance (10 gigohms) is used to record the peak voltage of 4.81 V (fig. S7), which should be close to the theoretical value of the open-circuit voltage (*39*). Since the PZT/LCE film has polarization, we inverse the connection of the upper and bottom surfaces to get the forward and backward current, which shows opposite profiles as expected, proving that the electric output comes from LCE/PZT itself (Fig. 2E). All results in Fig. 2 (C to E) have a PZT loading of 27.1 wt %. As the PZT loading is increased from 15.7 to 27.1, 42.7, and 60.1 wt %, respectively, the peak open-circuit voltage increases from 4.0 to 9.3, 21.3, and 27.4 mV, respectively. Simultaneously, the short-circuit current increases from 0.40 to 1.70 nA (Fig. 2F). At the highest loading of 60.1 wt %, *p* is calculated to be −3.30 nC cm^−2^ K^−1^, whose magnitude is 22% higher than that of the widely used flexible pyroelectric polymer PVDF (−2.70 nC cm^−2^ K^−1^) (*13*). Pure LCE shows no noticeable fluctuations in both current and voltage (fig. S8), confirming that the observed signals in LCE/PZT films are generated by the pyroelectric effect instead of thermal stress from LCE itself.

**Fig. 3.**
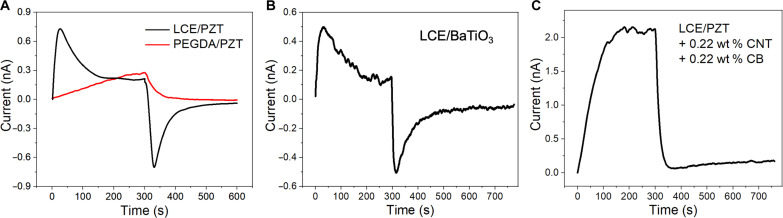
Short-circuit profiles of different composite materials. (**A**) Comparison between the polydomain LCE/PZT and PEGDA/PZT. (**B**) Polydomain LCE/BaTiO_3_. (**C**) Polydomain LCE/PZT composite with the addition of 0.22 wt % CNTs and 0.22 wt % CB.

The advantage of using LCE is further validated by replacing LCE with an isotropic elastomer, poly(ethylene glycol) diacrylate (PEGDA). PEGDA is chosen here because its diacrylate groups can be cross-linked with methacrylate groups on PZT NPs using the same thiol-acrylate Michael addition reactions (*34*). The short-circuit current of PEGDA/PZT is measured to be 0.28 nA, much smaller than 0.76 nA from the polydomain LCE/PZT (Fig. 3A). We note that the RM257/EDDT/PETMP LCE system is not special. Here, we tune their composition to have a relatively low Young’s modulus so that the film can remain flexible to generate sufficiently large stress even at a high PZT mass loading. When PZT NPs are changed to BaTiO_3_ NPs with a similar surface modification, the latter can be dispersed uniformly in the LCE matrix (see fig. S9). The polydomain LCE/BaTiO_3_ (27.1 wt %) has a peak current of 0.51 nA (Fig. 3B), smaller than that of the polydomain LCE/PZT (27.1 wt %). This is because BaTiO_3_ has a lower magnitude of *p* (−20.0 nC cm^−2^ K^−1^) compared to that of PZT (−26.8 nC cm^−2^ K^−1^) (*5*). To further increase the peak current, electrically conducting dopants can be added to reduce the resistance of the composite. For example, 0.22 wt % carbon nanotubes (CNTs) and 0.22 wt % carbon black (CB) are added to the polydomain LCE/PZT (27.1 wt %) system, achieving a peak current as high as 2.20 nA (Fig. 3C). CNTs enhance conductivity, while addition of CB makes the prepolymer processable instead of doubling CNT loadings as CNTs can substantially increase the viscosity (*21*).

### Pyroelectric output reliance on LCE alignment

**Fig. 4.**
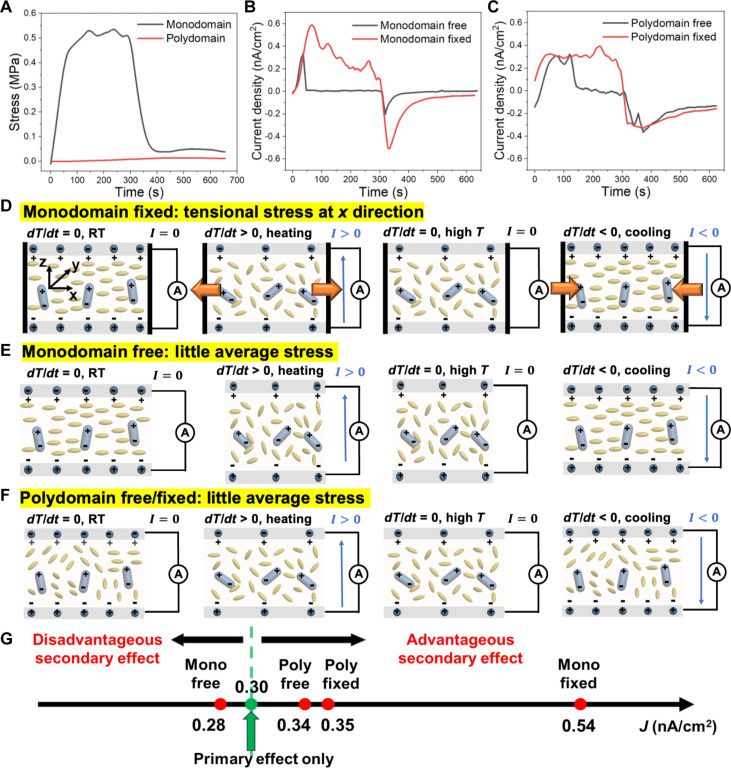
Effect of LCE alignment and boundary conditions on the output current of LCE/PZT composites with 27.1 wt % PZT NPs. (**A**) Stress profile of the monodomain LCE/PZT and polydomain LCE/PZT with the two ends fixed. (**B**) Short-circuit current density profiles of the monodomain LCE/PZT with the two ends fixed and two ends free. (**C**) Short-circuit current density profiles of the polydomain LCE/PZT with two ends fixed and two ends free. (**D** to **F**) Schematics of pyroelectricity of the LCE/PZT composite in a heating-cooling cycle with different LCE alignment and boundary conditions. (D) Monodomain LCE/PZT with two ends fixed, stretched at the longitudinal direction when heated. (E) Monodomain LCE/PZT with two ends free, showing little average stress when heated. (F) Polydomain LCE/PZT with little average stress when heated. RT, room temperature. (**G**) Illustration of the contributions of the secondary pyroelectric effect to the overall pyroelectricity depending on LCE alignment and boundary conditions.

Since the secondary pyroelectricity here relies on the intimate interactions between LCE and PZT NPs, both the alignment of LCE and the boundary conditions of the LCE/PZT film (fixed or not fixed at ends) can affect the pyroelectric output. When LCE/PZT is fixed at two ends, the monodomain sample shows a peak stress of 0.54 MPa, while the polydomain sample shows no apparent stress (Fig. 4A). It is expected that different stresses lead to different secondary pyroelectricity. Even at the same LCE alignment, whether the two ends of the film in the longitudinal direction are fixed or not can alter the output current. For the monodomain LCE/PZT, the peak current density is 0.54 nA/cm^2^ with two ends fixed but 0.28 nA/cm^2^ with two ends free (Fig. 4B). However, for the polydomain LCE/PZT, the peak current density does not vary much: 0.35 nA/cm^2^ (fixed) and 0.34 nA/cm^2^ (free) (Fig. 4C). To investigate the contributions from the primary and secondary pyroelectricity of the LCE/PZT system, we carry out a quantitative analysis as follows.

The primary pyroelectricity is related to the material’s intrinsic property, quantified with the primary pyroelectric coefficient, which has a negative value since electric displacement decreases at higher temperatures when dipoles become more isotropic. Therefore, the primary contribution of pyroelectricity in all four cases (polydomain versus monodomain, ends fixed or not) should be the same, which is 0.30 nA/cm^2^ from the calculation (Supplementary Text S1). Therefore, we focus on the differences in secondary pyroelectricity, which is piezoelectricity in essence, and whether the secondary effect works synergistically or counteracts with the primary effect when the film is subjected to different boundary conditions. First, we investigate the monodomain LCE/PZT. When the external electric field *E* is neglected, the strain-charge form of pyroelectricity becomes

$$\bar{D}=d\bar{\sigma }=\left[\begin{array}{cccccc} 0 & 0 & 0 & 0 & d_{15} & 0\\ 0 & 0 & 0 & d_{15} & 0 & 0\\ d_{31} & d_{31} & d_{33} & 0 & 0 & 0 \end{array}\right]\;\left[\begin{array}{c} {\bar{\sigma }}_{11}\\ {\bar{\sigma }}_{22}\\ {\bar{\sigma }}_{33}\\ {\bar{\sigma }}_{23}\\ {\bar{\sigma }}_{13}\\ {\bar{\sigma }}_{12} \end{array}\right]$$ (2)

where $\bar{D}$ is the average electric displacement, d is the piezoelectric coefficient, and $\bar{\sigma }$ is the average stress. While a soft matrix embedded with stiff inclusions has a complex stress state at the microscale because of the interfacial contributions and phase mismatches, the primary interest in pyroelectric applications is the overall electrical output (e.g., current and $\bar{D}$). Consequently, we use the average stress $\bar{\sigma }$ of the entire composite structure in this estimation approach without modeling the intricate local stress field **σ**(***x***, *t*). Since the output current in the *z* direction is measured, the third component of the average electric displacement, ${\bar{D}}_{3}$, can be described by

$${\bar{D}}_{3}=d_{31}{\bar{\sigma }}_{11}+d_{31}{\bar{\sigma }}_{22}+d_{33}{\bar{\sigma }}_{33}$$ (3)

Plugging in the parameters for PZT materials, we obtain

$${\bar{D}}_{3}=-2.74{\bar{\sigma }}_{11}-2.74{\bar{\sigma }}_{22}+5.93{\bar{\sigma }}_{33}\left(\times 10^{-10}\;C/N\right)$$ (4)

For a monodomain LCE/PZT film fixed at two ends in the *x* direction (Fig. 4D), the local stresses in the *y* and *z* directions tend to cancel out since the film can freely deform in these directions. Therefore, compared to ${\bar{\sigma }}_{11}$, the average stresses ${\bar{\sigma }}_{22}$ and ${\bar{\sigma }}_{33}$ are approximately zero. Thus, we can estimate the secondary pyroelectric coefficient by

$${\bar{D}}_{3}\approx -2.74{\bar{\sigma }}_{11}\left(\times 10^{-10}\;C/N\right)$$ (5)

$$p_{2}=\frac{\partial {\bar{D}}_{3}}{\partial T}=-2.74\frac{\partial {\bar{\sigma }}_{11}}{\partial T}\left(\times 10^{-10}\;C/N\right)$$ (6)

When the LCE/PZT film is heated with a positive value of $\frac{\partial \sigma_{11}}{\partial T}$, a negative secondary pyroelectric coefficient will enhance the output current as seen in Fig. 4D. Now, we conclude that the output current density is higher than the primary contribution, 0.30 nA/cm^2^. Theoretical calculations give the estimated output current density of ~0.50 nA/cm^2^ (with fixed ends), which matches with experimental results of 0.54 nA/cm^2^ (Supplementary Text S1). The slightly higher value from experiments may come from the nonuniform stress distributions at the microscale.

This approach can be extended to other boundary conditions, for example, a monodomain LCE/PZT film with all ends free (Fig. 4E). Theoretically, the average stresses from all directions are close to zero, although experimentally, it is difficult to meet this. Given that ${\bar{D}}_{3}\approx 0$ and $p_{2}\approx 0$, the output current density should be close to the pure primary contribution, 0.30 nA/cm^2^, which matches with the experimental result, 0.28 nA/cm^2^.

The discussion on the monodomain LCE/PZT can be partly applied to the polydomain LCE/PZT featured by the interactions between the LCE and PZT. In a polydomain phase, since each LC domain is oriented randomly against each other, the overall interactions are canceled out (Fig. 4F), resulting in little overall secondary pyroelectricity. Therefore, the output current density for the polydomain LCE/PZT should be around 0.30 nA/cm^2^, which is purely the contribution from the primary pyroelectricity, regardless of whether the film is fixed or not. The current density obtained from experiments (0.34 to 0.35 nA/cm^2^) is slightly higher than the calculation, which may be attributed to residual orders in the polydomain film.

To sum up, the secondary contribution to pyroelectricity is dependent on LCE alignment and the boundary conditions (Fig. 4G). The monodomain LCE/PZT under fixed boundary conditions shows the highest secondary effect that enhances the overall pyroelectricity. The polydomain LCE/PZT shows weak secondary effects according to experiments. Nonfixed monodomain LCE/PZT has reduced overall pyroelectricity. We note that all the discussions above are based on the composite films with 27.1 wt % PZT loading, and the highest possible PZT loading is 42.7 wt % instead of 60.1 wt % for the monodomain LCE/PZT since the samples at higher PZT loadings crack when stretched. Under fixed boundary conditions, the monodomain LCE/PZT (42.7 wt % PZT) shows a peak output current of 2.81 nA and a voltage of 6.23 V (fig. S10), yielding a pyroelectric coefficient of −4.01 nC cm^−2^ K^−1^, whose magnitude is 49% higher than that of the widely used flexible pyroelectric polymer PVDF (−2.70 nC cm^−2^ K^−1^) (*13*).

**Fig. 5.**
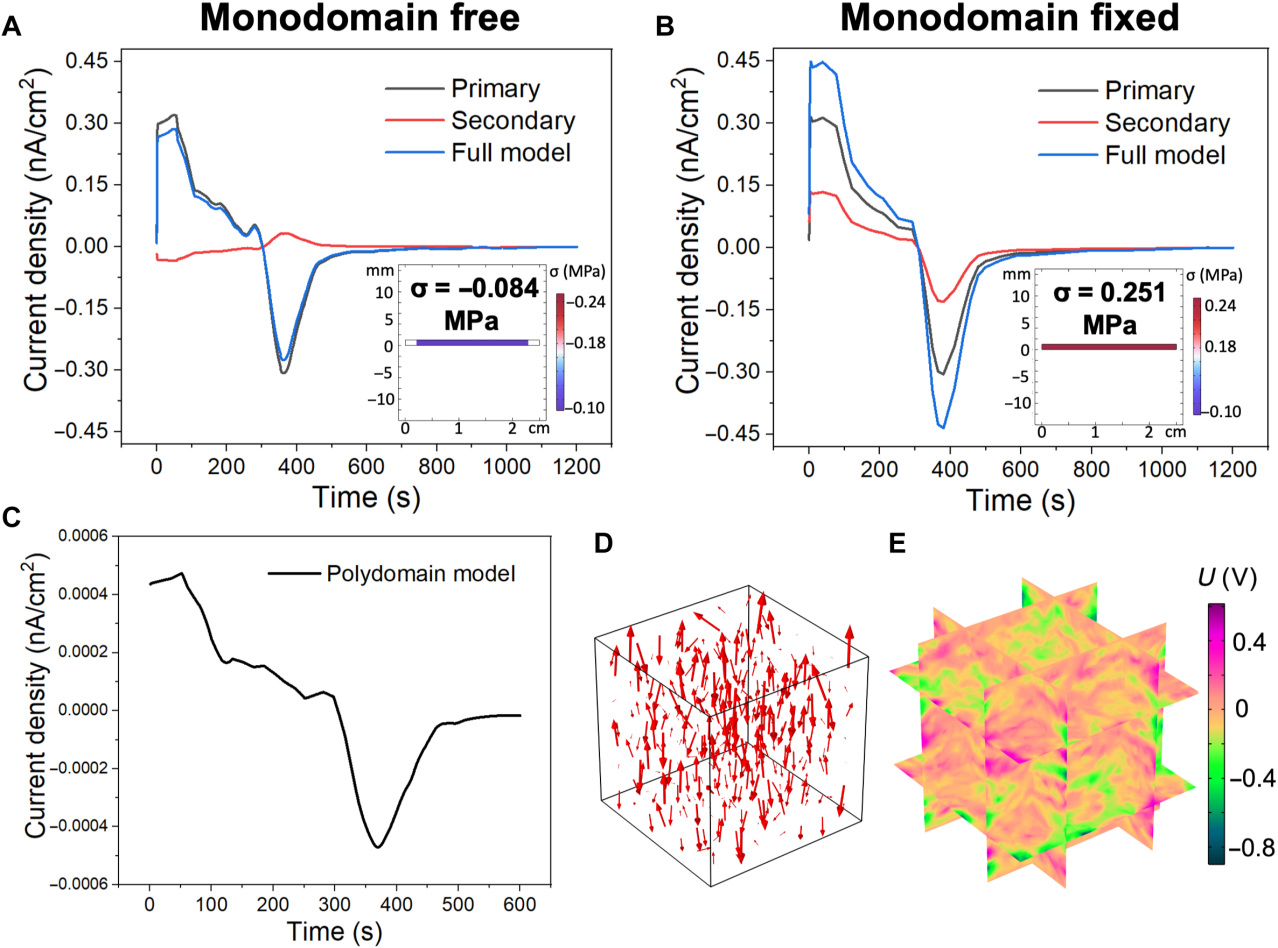
Finite element simulations of the LCE/PZT composite with 27.1 wt % PZT NPs with different LCE alignment and boundary conditions. (**A** and **B**) Simulated short-circuit current density profile of the monodomain LCE/PZT with (A) two ends free and (B) two ends fixed, showing the primary effect, secondary effect, and the whole model. Inset: simulated stress distributions of the LCE/PZT films at the maximum stress. (**C**) Simulated short-circuit current density profile of the polydomain LCE/PZT, showing the secondary effect only. (**D** and **E**) Simulated current density (D) and electric potential (E) profiles of the polydomain LCE/PZT at the peak output current.

To verify the respective contribution to the secondary pyroelectricity by LCE alignment and the boundary conditions, we conduct finite element simulations by COMSOL Multiphysics (Supplementary Text S2). For the monodomain LCE/PZT, the contributions from primary pyroelectricity and secondary pyroelectricity are successfully decoupled (Supplementary Text S3). The simulated primary pyroelectric current density is 0.30 nA/cm^2^ for both free and fixed boundary conditions. Under a free boundary condition, the peak stress is −0.084 MPa, and the simulated secondary pyroelectric current density is −0.02 nA/cm^2^, giving an overall pyroelectric current density of 0.28 nA/cm^2^ (Fig. 5A). Under a fixed boundary condition, the peak stress is 0.251 MPa, and the simulated secondary pyroelectric current density is 0.14 nA/cm^2^, giving an overall pyroelectric current density of 0.44 nA/cm^2^ (Fig. 5B). The simulation results match well with experimental results. For the polydomain LCE/PZT, 64,000 domains are simulated, with each domain sized 10 by 10 by 10 μm. Each domain’s orientation is described by a random unit vector. Therefore, the thermal contraction of each domain occurs in different random directions. The simulated peak current density from the secondary pyroelectricity is only 0.0005 nA/cm^2^, which is three magnitudes lower than that from the primary pyroelectricity, 0.30 nA/cm^2^ (Fig. 5C). Both the spatial current density distribution and electric potential distribution are random everywhere (Fig. 5, D and E), which further proves the little contribution of secondary pyroelectricity in the polydomain LCE/PZT.

Besides LCE alignment, the thermomechanical properties of the LCE matrix, including the Young’s modulus and the thermal expansion coefficient, could affect the secondary pyroelectricity, thus the overall pyroelectric output when the ends of the monodomain LCE/PZT film are fixed. We compare the mechanical properties of six different LCE recipes, and their pyroelectric output from both experiments and simulations is summarized in table S2 (films fabricated using recipe #3 are mainly used in the reported studies). Recipes #3 and #4 with relatively high Young’s moduli (1.70 to 2.19 MPa) show a similar pyroelectric current density of 0.54 nA/cm^2^, larger than those of recipes #1 and #2 (0.46 to 0.47 nA/cm^2^), which have relatively lower Young’s moduli (0.66 to 1.05 MPa). The thermal expansion coefficient is not substantial here (see detailed discussion in Supplementary Text S4). Generally, the contribution by the thermomechanical properties of the LCE matrix to pyroelectricity is minor compared to that by the LCE alignment.

### Application demonstration

**Fig. 6.**
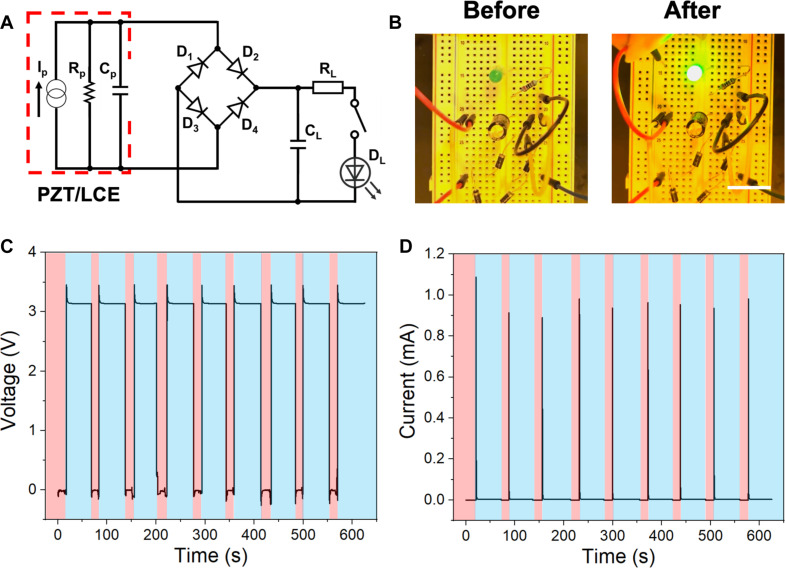
Demonstrations of the LCE/PZT pyroelectric energy harvester. (**A**) Electric circuit to charge a capacitor and then light an LED using the LCE/PZT heat harvester. (**B**) Pictures of the green LED before (left) and after (right) being powered by the LCE/PZT energy harvester. Scale bar, 2 cm. (**C** and **D**) Voltage (C) and current (D) profiles of the LED during nine cycles, where the pink areas indicate the capacitor charging, and the blue areas are for LED powering.

To demonstrate the potential application of the LCE/PZT composite film as a heat energy harvester, we connect it to a simple circuit composed of capacitors, a rectifier, and a green LED (Fig. 6A). Our LCE/PZT film can charge a 0.22-μF capacitor in 15 s with a torch lamp, and the capacity is connected to a green LED for 55 s. It powers the LED for 5 s with a peak voltage of 3.4 V and a peak current of 1.1 mA (Fig. 6B). To verify the robustness of the LCE/PZT thermal energy harvester, we perform nine continuous cycles (movie S1). The voltage and current of the LED during cycling tests are recorded, with the red areas labeled for charging the capacitor and the green areas labeled for lighting the LED in Fig. 6 (C and D). In each cycle, the peak voltage is stabilized at 3.4 V, and the peak current is in the range of 0.9 to 1.1 mA, with no obvious degradation observed. The peak power is 3.75 mW, which outcompetes most pyroelectric energy harvesters with a power typically on the order of microwatt scale. The heat energy conversion efficiency is 0.021%, which seems low since heat is very lossy, but it is better than most pyroelectric energy harvesters with energy conversion efficiency too low to be reported (fig. S11 and Supplementary Text S5).

## DISCUSSION

In summary, we fabricate an LCE/PZT pyroelectric energy harvester with greatly enhanced pyroelectricity because of the secondary pyroelectricity generated from LCE thermal stress. The effect of secondary pyroelectricity on the overall pyroelectric output is dependent on LCE alignment and boundary conditions. The monodomain LCE/PZT under fixed boundary conditions exhibits the highest secondary pyroelectric effect, with the output current density increased from 0.30 nA/cm^2^ (primary pyroelectricity) to 0.54 nA/cm^2^, which is verified by both experiments and simulations. The fixed monodomain LCE/PZT film (42.7 wt % PZT) shows an output current of 2.81 nA and a voltage of 6.23 V at a maximum heating rate of 0.20 K s^−1^, corresponding to a pyroelectric coefficient *p* of −4.01 nC cm^−2^ K^−1^, whose magnitude is 49% higher than that of the widely used flexible pyroelectric polymer PVDF (*p*, −2.70 nC cm^−2^ K^−1^) (*13*). The LCE/PZT composite can serve as a power source that harvests the heat to drive flexible electronics such as LEDs, digital watches, and sensors.

It is noted that the output power of the LCE/PZT pyroelectric energy harvester is still limited to support high-power appliances. This is mainly because of the high resistance of LCE/PZT. The output pyroelectric properties can be further improved by adding ferroelectric or electrically conductive fillers. In addition, PZT contains lead, which is not environmentally friendly. When replacing PZT with BaTiO_3_, a similar output performance is obtained, suggesting that the mechanism of generating pyroelectricity in LCE-pyroelectric composite is universal. Therefore, other environmentally friendly materials can be applied to this LCE composite system in future studies. We envision that our LCE/PZT composites will not only open application avenues for LCE composites but also inspire designs where nanofillers and the LCE matrix mutually enhance each other’s properties.

## MATERIALS AND METHODS

### Experimental design

The study fabricated LCE/PZT composites, which included the surface modification of PZT NPs and the fabrication of the LCE/PZT film. Steps were taken to achieve different alignments of LCE, including the monodomain and polydomain. Characterizations were conducted to reveal the molecular bonding, particle distribution, mechanics, and thermal properties of the LCE/PZT film. We used a torchlamp to heat the LCE/PZT film for testing pyroelectric properties, and the output current and voltage were measured by a multimeter. Please see the following sections for more details.

#### Materials

PZT (APC 850) and BaTiO_3_ were purchased from APC Materials. TMSPMA (≥97%), DPA (99%), EDDT (95%), PETMP (>95%), PEGDA (number-average molecular weight, 700 g/mol), and 1,1′-azobis(cyclohexanecarbonitrile) (98%) were purchased from Sigma-Aldrich. Ethanol (99.5%, ACS reagent, absolute, 200 proof), hydrochloride acid (HCl, 37%), and acetic acid (Glacial ACS) were purchased from Thermo Fisher Scientific. The LC monomer RM257 (>95%) was purchased from Wilshire Technologies Inc. All chemicals were used without further purification. Ultrapure water produced by a Milli-Q Integral 5 system was used in all experiments.

#### Surface functionalization of the PZT NPs

The synthesis was conducted following the literature with modification (*40*). Briefly, PZT NPs were ball milled to an average size of <1 μm (see SEM image in fig. S4). Then, 3.2 g of PZT NPs was dispersed in a 200-ml ethanol solution with 4 ml of TMSPMA and a 125-μl acetic acid aqueous solution (acetic acid:H_2_O = 1:9 volume ratio). The mixture was sonicated for 24 hours. The final product was washed twice with ethanol to remove any unreacted TMSPMA, followed by centrifugation at 2500 rpm for 15 min. After the ethanol solution was decanted, the PZT NPs were collected and dried in a 60°C oven overnight before use.

#### Fabrication of the LCE/PZT and PEGDA/PZT films

RM257 (5 g) was dissolved in 1.55 g of toluene by heating the mixture in a 90°C oven for 15 min. The saturated solution was cooled to room temperature. Then, 0.266 g of *tetra*-thiol cross-linker PETMP, 1.276 g of dithiol chain extender EDDT, 0.15 g of thermal initiator 1,1′-azobis(cyclohexanecarbonitrile), and a desired amount of TMSPMA-modified PZT NPs (1.25, 2.5, 5, or 10 g) were added. The mixture was put in a 60°C oven for another 15 min to complete mixing. The catalyst DPA (0.81 g; 2 wt % in toluene) was added into the mixture and stirred slowly after cooling to room temperature. The mixture was vacuumed for 1 min if air bubbles were present. Afterward, the viscous solution was poured into a rectangular mold using 1-mm-thick VHB tape (3M) as the spacer and left for 12 hours to finish the thiol-arylate Michael addition reactions. The resulting LCE/PZT gel was put into a 60°C oven for 18 hours to remove the solvent. The polydomain sample was obtained by directly cross-linking the LCE/PZT gel in a 90°C oven for 18 hours (*41*). For the monodomain sample, the LCE/PZT film was stretched at a 100% strain, fixed on a glass slide with two clippers, and then cross-linked in a 90°C oven for 18 hours (*29*). For the fabrication of PEGDA/PZT, 5.95 g of PEGDA and 2.9 g of PZT were used while keeping all the other procedures the same. The as-fabricated samples were poled under 1 kV mm^−1^ at 120°C for 4 hours between two indium tin oxide glasses. The samples, after poling, were assembled using copper tapes as electrodes and were further encapsulated with Kapton tapes.

#### Characterization

SEM (JEOL 7500F HRSEM) was operated at 5 kV to characterize the size and size distribution of PZT NPs, as well as the dispersity of the PZT or BaTiO_3_ NPs in the LCE matrices. FTIR spectroscopy was performed using a Thermo Fisher Scientific 6700 FTIR spectrometer in the transmission mode using compressed KBr pellets. An upright motorized microscope (BX61, Olympus) in the transmission mode equipped with cross-polarizers was used to verify the monodomain alignment of LCE films. For the measurement of the pyroelectric performance, a high-power light bulb (TL2 600-Watt, Smith-Victor) was used to change the temperature of the film, and the distance between the film and the light bulb was maintained at 20 cm to obtain a constant heating rate. The temperature was monitored by a handheld thermal imager (Fluke TiS75+). The open-circuit voltage and the short-circuit current were measured by a Keithley DMM6500. Differential scanning calorimetry tests were performed on the TA Instruments Q2000. The LCE/PZT film (27.1 wt % PZT) was cut into small pieces and placed into hermetic aluminum pans. Samples were heated and cooled at a ramping rate of 10°C/min under N_2_ for two cycles, from −50° to 150°C, and data from the second cycle were reported here.

### Statistical analysis

All graphs were drawn by Origin 2024b. For the graph with error bars (Fig. 2F), at least six data points were gathered, while the standard deviations were calculated and reflected on the error bars. For all the other figures, the experiments were conducted at least twice with consistent results, and the results from the last experiment were reported.

## Supplementary Materials

### The PDF file includes:

Supplementary Texts S1 to S5 Figs. S1 to S11 Tables S1 and S2 Legend for movie S1 References
