# Supplementary material for: Flexible pyroelectric energy harvesters from nanocomposites of liquid crystal elastomers/lead zirconate titanate nanoparticles
Source: Sci Adv. 2025 Feb 12;11(7):eadt6136. doi: 10.1126/sciadv.adt6136 (PMC11817937; doi:10.1126/sciadv.adt6136)
Supplement: Supplementary file 1 — Supplementary Texts S1 to S5 Figs. S1 to S11 Tables S1 and S2 Legend for movie S1 References [file sciadv.adt6136_sm.pdf]

Science Advances
AAAS

Supplementary Materials for

Flexible pyroelectric energy harvesters from nanocomposites of liquid crystal elastomers/lead zirconate titanate nanoparticles

Shangsong Li *et al.*

Corresponding author: Shu Yang, shuyang@seas.upenn.edu

Sci. Adv. **11**, eadt6136 (2025)
DOI: 10.1126/sciadv.adt6136

The PDF file includes:

Supplementary Texts S1 to S5
Figs. S1 to S11
Tables S1 and S2
Legend for movie S1
References

Other Supplementary Material for this manuscript includes the following:

Movie S1

Supplementary Text

Supplementary Text S1. Theoretical calculations of LCE/PZT

Depending on whether LC mesogens are aligned uniformly or not and whether the LCE/PZT films are fixed on two ends or not, they exhibit very different deformation behaviors and stress distributions in response to thermal stimuli, which influences the secondary effect, where temperature changes induce strain and generate charges. The primary pyroelectricity only relates to the primary pyroelectric coefficient (a negative value since electric displacement decreases at higher temperatures when dipoles become more chaotic). To understand the respective contributions of primary pyroelectricity to secondary pyroelectricity, we perform calculations to estimate the primary and secondary pyroelectric coefficients.

From the discussions in the main text, we know that with two ends fixed, the expression of the secondary pyroelectric coefficient p_2 can be described as

$$p_2 = -2.74 \frac{\partial \bar{\sigma}_{11}}{\partial T} (\times 10^{-10}\ \mathrm{C/N}) \tag{S1}$$

Where $\bar{\sigma}_{11}$ is the average stress in the longitudinal direction. When heated above the nematic to isotropic transition temperature, T_{NI}, LCE transforms from monodomain into the isotropic phase. With two ends fixed, contraction above T_{NI} is not allowed in the *x*-direction, thus, leading to a tensile stress imposed to the film to counteract the tendency of shrinkage (Fig. 4d). Here, the sign of the average stress should be positive,

$$\bar{\sigma}_{11} > 0 \tag{S2}$$

$$p_2 = -2.74 \frac{\partial \bar{\sigma}_{11}}{\partial T} (\times 10^{-10}\ \mathrm{C/N}) < 0 \tag{S3}$$

Thus, for a fixed LCE/PZT film, the secondary pyroelectric effect enhances the overall pyroelectric effect. This contrasts with almost all ferroelectrics (including PZT), where p_2 always has a opposite sign from p_1. Next, we investigate whether the contribution of secondary effect is large enough to make a difference. We first need to know $\frac{\partial \bar{\sigma}_{11}}{\partial T}$, which is difficult to measure directly from experiments as it changes with time. For proof-of-concept, we take the maximum value to estimate, which occurs at the initial heating stage with a roughly linear relationship. We measure the stress-time relationship $\frac{\partial \bar{\sigma}_{11}}{\partial t}$, and the temperature-time relationship $\frac{\partial T}{\partial t}$, separately. According to the chain rule of differentiation,

$$\frac{\partial \bar{\sigma}_{11}}{\partial T} = \frac{\frac{\partial \bar{\sigma}_{11}}{\partial t}}{\frac{\partial T}{\partial t}} \tag{S4}$$

For the temperature profile (Fig. 2a), at each initial heating stage, the time is ~100 s, and the temperature change is ~20 K. Therefore, we have the approximation

$$\frac{\partial T}{\partial t} \approx 0.2\ \mathrm{K/s} \tag{S5}$$

For the stress profile of the monodomain LCE/PZT (Fig. 4a), at the initial heating stage, the time is ~60 s, and the stress change is ~0.43 MPa. Therefore, p_2 can be calculated by

$$\frac{\partial \bar{\sigma}_{11}}{\partial t} \approx 0.0072\ \mathrm{MPa/s} \tag{S6}$$

$$\frac{\partial \bar{\sigma}_{11}}{\partial T} = \frac{\frac{\partial \bar{\sigma}_{11}}{\partial t}}{\frac{\partial T}{\partial t}} \approx \frac{0.0072\ \mathrm{MPa/s}}{0.2\ \mathrm{K/s}} = 0.036\ \mathrm{MPa/K} \tag{S7}$$

$$p_2 = -2.74 \frac{\partial \sigma_{11}}{\partial T} \left(\times 10^{-10}\,\frac{\mathrm{C}}{\mathrm{N}}\right) \approx -2.74 \times 10^{-10} \times 0.036 \times 10^{6}\,\frac{\mathrm{C}}{\mathrm{m^2 \cdot K}} \tag{S8}$$

$$p_2 \approx -0.986\,\frac{\mathrm{nC}}{\mathrm{cm^2 \cdot K}} \tag{S9}$$

Now, let us estimate the contribution from the primary pyroelectric effect. For pure PZT, we have

$$p_{total} = -26.8\,\frac{\mathrm{nC}}{\mathrm{cm^2 \cdot K}} \tag{S10}$$

When the concentration of PZT in the LCE/PZT composite is 27.1 wt%, or 5.6 vol%, the pyroelectric effect should be normalized by volume fraction since PZT is pyroelectric, but LCE is not. Therefore, we estimate the primary pyroelectric coefficient p_1

$$p_1 \approx -26.8 \times 5.6\% = -1.501\,\frac{\mathrm{nC}}{\mathrm{cm^2 \cdot K}} \tag{S11}$$

It suggests that the secondary effect $-0.986\,\frac{\mathrm{nC}}{\mathrm{cm^2 \cdot K}}$ has the same magnitude compared to the primary effect $-1.501\,\frac{\mathrm{nC}}{\mathrm{cm^2 \cdot K}}$. Therefore, the secondary effect could substantially enhance the overall pyroelectric effect. For the monodomain LCE/PZT with fixed ends, the total pyroelectric coefficient is

$$p_{total} = p_1 + p_2 \approx -2.487\,\frac{\mathrm{nC}}{\mathrm{cm^2 \cdot K}} \tag{S12}$$

which corresponds to the calculated current density

$$J_{mono,fixed} = |p \frac{\partial T}{\partial t}| \approx 0.497 \approx 0.50\ \mathrm{nA/cm^2} \tag{S13}$$

This is close to the experimental result of 0.54 nA/cm^2.

Supplementary Text S2. Finite element simulation of LCE/PZT

Ferroelectric-type pyroelectric materials demonstrate a primary pyroelectric effect when there is a change in temperature while the shape and volume remain constant. Nevertheless, this effect is often accompanied by a secondary effect that arises from thermal expansion and piezoelectricity, making it challenging to differentiate them in experiments. To quantify their respective contributions, it is necessary to perform finite element method (FEM) simulations using tools such as COMSOL Multiphysics. The Heat Transfer in Solids module simulates the heating and cooling processes of the LCE/PZT composite for a specific temperature boundary condition $T(t)$.

$$d_z\rho C_p\frac{\partial T}{\partial t}+d_z\rho C_p\mathbf{u}\cdot\nabla T+\nabla\cdot\left(-d_z k\nabla T\right)=0 \qquad \text{(S14)}$$

The simulations are conducted assuming zero strain in the out-of-plane direction, which is the case of LCE upon heated above T_{NI}. Here, d_z is the width of the samples, ρ is the material density, C_p is the heat capacity, and $\mathbf{u}$ is the velocity field. The Pyroelectricity (pye) multiphysics interface can then be used to calculate the current density resulting from the temperature change dT/dt, according to

$$\mathbf{J}=\frac{d\mathbf{P}_e}{dt}=\frac{d\left[T(t)-T_0\right]\mathbf{p}_{ET}}{dt} \qquad \text{(S15)}$$

where $\mathbf{J}$ represents the current flux, $\mathbf{P}_e$ stands for the material polarization, T_0 is the initial temperature, and $\mathbf{p}_{ET}$ is the total pyroelectric coefficient. Specifically, $\mathbf{p}_{ET}$ is defined as $\mathbf{p}_{ET}=\mathbf{p}_{ES}+\mathbf{e}_{ES}\boldsymbol{\alpha}$, where $\mathbf{p}_{ES}$ denotes the primary pyroelectric coefficient measured at constant strain. The second term in the equation is generally called the secondary coefficient, with piezoelectric coupling matrix $\mathbf{e}_{ES}$ and coefficient of thermal expansion $\boldsymbol{\alpha}$. The Electrostatics (es) and Electrical Circuit (cir) modules establish fundamental electrical laws and output signals. The Solid Mechanics (solid) and Thermal Expansion (te) modules are used to calculate the thermal strain $\mathbf{s}_{th}=\boldsymbol{\alpha}[T(t)-T_0]$ caused by the temperature change, as well as the corresponding thermal stress $\boldsymbol{\sigma}_{th}=\mathbf{c}_E\boldsymbol{\varepsilon}_{th}$, where $\mathbf{c}_E$ is the stiffness tensor of the material. Then, Piezoelectric Effect (pze) interface can be implemented to calculate the piezoelectricity-induced polarization based on the linear piezoelectric constitutive equations,

$$\begin{aligned}\mathbf{S}&=\mathbf{s}_E\boldsymbol{\sigma}+\mathbf{d}^T\mathbf{E}\\ \mathbf{D}&=\mathbf{d}\boldsymbol{\sigma}+\boldsymbol{\varepsilon}_T\mathbf{E}\end{aligned} \qquad \text{(S16)}$$

This equation expresses the relation between strain $\mathbf{S}$, stress $\boldsymbol{\sigma}$, electric field $\mathbf{E}$, and electric displacement field $\mathbf{D}$, where $\mathbf{s}_E$ represents the elastic compliance tensor under constant electric field, defined as $\mathbf{s}_E=\mathbf{c}_E^{-1}$, $\mathbf{d}$ is the piezoelectric constant tensor, and $\boldsymbol{\varepsilon}_T$ denotes the dielectric constant tensor.

Supplementary Text S3. FEM simulations for a monodomain LCE/PZT film

A classical approach to decouple the primary and secondary pyroelectric effects is to simulate the short-circuit currents **I** generated by the material under the fixed-end and free-end boundary conditions, respectively. Different boundary conditions provide different states of stress and strain inside the material, resulting in different secondary currents $\mathbf{I}_{\mathrm{sec}}$, but the primary currents $\mathbf{I}_{\mathrm{prim}}$ are identical because $\mathbf{I}_{\mathrm{prim}}$ is only related to the rate of change of temperature with time (dT/dt). The total current for the two boundary conditions ($\mathbf{I}_{\mathrm{fix}}$ and $\mathbf{I}_{\mathrm{free}}$) can be calculated by

$$\mathbf{I_{fix}} = \mathbf{I_{prim}} + \mathbf{I_{sec,fix}} = \mathbf{p_1} A \frac{dT}{dt} + \frac{d(\mathbf{dS_{fix}})}{dt} \tag{S17}$$

$$\mathbf{I_{free}} = \mathbf{I_{prim}} + \mathbf{I_{sec,free}} = \mathbf{p_1} A \frac{dT}{dt} + \frac{d(\mathbf{dS_{free}})}{dt} \tag{S18}$$

where A is the surface area. $\mathbf{I}_{\mathrm{fix}}$ and $\mathbf{I}_{\mathrm{free}}$ can be measured experimentally, and the stress states under fixed-end and free-end conditions $\mathbf{S}_{\mathrm{fix}}$ and $\mathbf{S}_{\mathrm{free}}$ can be quantified by numerical simulations. Therefore, in Eqs. S17 and S18, only $\mathbf{p_1}$ and **d** are the unknowns that can be solved, or otherwise, it will be challenging to measure experimentally. Accordingly, we can find out the respective contributions of $\mathbf{I}_{\mathrm{prim}}$ and $\mathbf{I}_{\mathrm{sec}}$. The current density **J** is calculated by dividing **I** over the film area. The total, primary, and secondary current densities under fixed-end and free-end conditions are shown in Figures 4a-b. It is clear that $\mathbf{I}_{\mathrm{prim}}$ is the same, whether fixed-end or free-end conditions, and proportional to dT/dt. However, changing the boundary conditions alters the stress state inside the material and produces different secondary currents $\mathbf{I}_{\mathrm{sec}}$. The stresses and deformations of monodomain LCE/PZT under the two boundary conditions are shown in the inset of Figures 4a-b. The greater von Mises stresses make the secondary effect more prominent in the fixed-end condition than in the free-end condition. More importantly, the primary and secondary effects are synergistic in the fixed-end condition, and their currents are in the same direction, whereas in the free-end condition, the two effects are canceling of each other due to the change in the direction of the stress vector **S**.

According to the simulation results, the peak total current density generated from the monodomain LCE/PZT film under the fixed-end and free-end conditions are 0.44 nA and 0.28 nA, respectively. Compared with the experimental results, the deviations are 18.5% and 0%, respectively.

Supplementary Text S4. The effect of LCE matrix mechanical properties on the pyroelectric performance of LCE/PZT composites

Whether the mechanical property of the LCE matrix affects the pyroelectric performance depends on the LCE alignment. For polydomain LCE/PZT composites, the mechanical property of the LCE matrix nearly has no influence on the pyroelectric performance. That is because they have little secondary pyroelectricity due to the little thermal stress generated in polydomain LCEs. Therefore, the pyroelectric output of polydomain LCE/PZT composites all comes from the primary pyroelectricity, which only relies on the mass loading of PZT. Similarly, for monodomain LCE/PZT composites with free boundary conditions, the secondary pyroelectricity is nearly zero due to the negligible average stress.

For monodomain LCE/PZT composites with the two longitudinal ends fixed, the mechanical property of the LCE matrix can affect the pyroelectric performance, since the secondary pyroelectricity makes a difference now. A higher Young's modulus can give a higher thermal stress, thus enhancing the secondary pyroelectricity. A higher thermal expansion coefficient gives a larger thermal strain, which leads to enhanced thermal stress and secondary pyroelectricity. However, in real cases, the Young's modulus and thermal expansion coefficient change simultaneously, making it difficult to decouple their separate effect. Therefore, to test the effect of LCE mechanical properties on the pyroelectric performance in real situations, we fabricated monodomain LCE/PZT films (27.1 wt% PZT) with different molar ratios of PETMP to EDDT, or acrylate to thiol groups. we then measured their Young's moduli and their thermal expansion coefficients. Subsequently, we measured their pyroelectric currents, and conducted FEM simulations using the measured parameters as new input to verify the experiments. The results are summarized in Table S2. Recipe #3 is the composition used in the main studies.

When the molar ratio of EDDT to PETMP was low (e.g., 9.6 in recipe #5, lower than 13 in recipe #3), the relatively high amount of crosslinker PETMP would make the polymer brittle, which fractured during the two-step crosslinking process. When the molar ratio of acrylates to thiols was too high (e.g., 13 in recipe #6), the degree of polymerization was too low to form a film after the Michael addition reaction, thus the two-step crosslinking could not proceed. Recipes #1-#4 gave monodomain films successfully. When the molar ratio of EDDT to PETMP was the highest, 16.3, the lowest Young's modulus was obtained, 0.66 MPa, and a reduced secondary pyroelectricity was observed. Meanwhile, a lower Young's modulus led to a higher absolute value of thermal expansion coefficient, which enhanced the secondary pyroelectricity. Therefore, the Young's modulus and thermal expansion coefficient counteract with each other regarding the secondary pyroelectricity. This proves again that they cannot be decoupled.

For the pyroelectric current density, recipes #3 and #4 give the highest experimental results of 0.54 nA/cm^2, while recipes #1 and #2 show a little lower output, 0.46 and 0.47 nA/cm^2, respectively. Simulation results show a similar trend. We note that compared to #1 and #2, #3 and #4 have higher Young's moduli, which should enhance pyroelectric output, and lower the thermal expansion coefficients, and thus decreasing pyroelectric output. Experimentally, we observed that the highest current density was obtained from recipes #3 and #4. Therefore, we can conclude that the effect from the Young's modulus dominates.

When the Young's modulus was either too high (recipe #5) or too low (recipe #6), we failed to fabricate the monodomain films. The Young's modulus of 2.19 MPa in the recipe #4 was close to the upper limit. Moreover, since recipe #3 gave a little smaller Young's modulus

(1.70 MPa), while achieving the similar pyroelectric output as the #4, it suggested that the effect of Young's modulus on pyroelectricity plateaued when it went above a certain value. For future flexible electronics applications, the LCE/PZT films should have both high pyroelectric output and good flexibility. Therefore, the recipe #3 was chosen in the study reported here.

Supplementary Text S5. Calculation of the energy conversion efficiency

We use the LCE/PZT film with 27.1 wt% PZT (length 25 mm, width 10 mm, thickness 1.0 mm), and the average sample mass (m) is 0.356 g. DSC measurements indicate that the heat capacity of the LCE/PZT, c, is 1.95 J/g·K (Fig. S6). In each cycle, the charging stage lasts for 15 s, with the temperature of LCE/PZT increasing from 298 K to 308 K due to torch lamp irradiation. The heat absorption can be expressed as

$$Q = cm\Delta t \tag{S19}$$

where Δt is temperature change. Accordingly, the heat absorbed, or the input energy in one cycle, is $Q = 6.94\,J$.

The output energy is the electric work of the LED light during the discharging stage, lasting for 55 s. In each cycle, we have a pattern of voltage $U(i)$ (Fig. S11a) and a pattern of current $I(t)$ (Fig. S11b). Since the electric power P can be expressed by $P = UI$, we can get the function $P(t)$ by involution of the $U(t)$ and $I(t)$ curves (Fig. S11c).

The electric work can be calculated by integration of the $P(t)$ function

$$W = \int_0^t P(t)\,dt \tag{S20}$$

Plug in the $P(t)$ function curve above, we obtain $W = 1.46$ mJ.

Therefore, the energy conversion efficiency

$$\eta = \frac{W}{Q} = \frac{1.46\ \text{mJ}}{6.94\ \text{J}} = 0.021\ \% > 0.01\% \tag{S21}$$

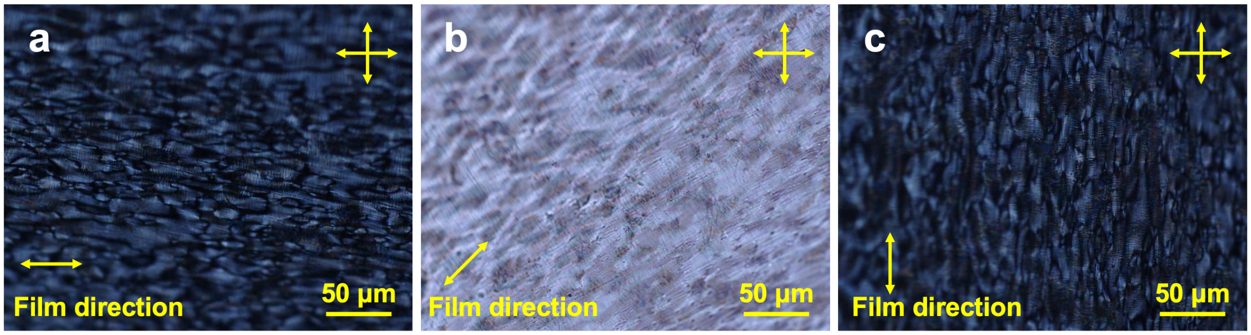

Fig. S1. Polarized optical microscope (POM) images of the monodomain LCE under the crossed polarizers. Images are taken at LCE film longitudinal direction, which is (a) parallel, (b) 45° to, and (c) perpendicular to one of the polarizers.

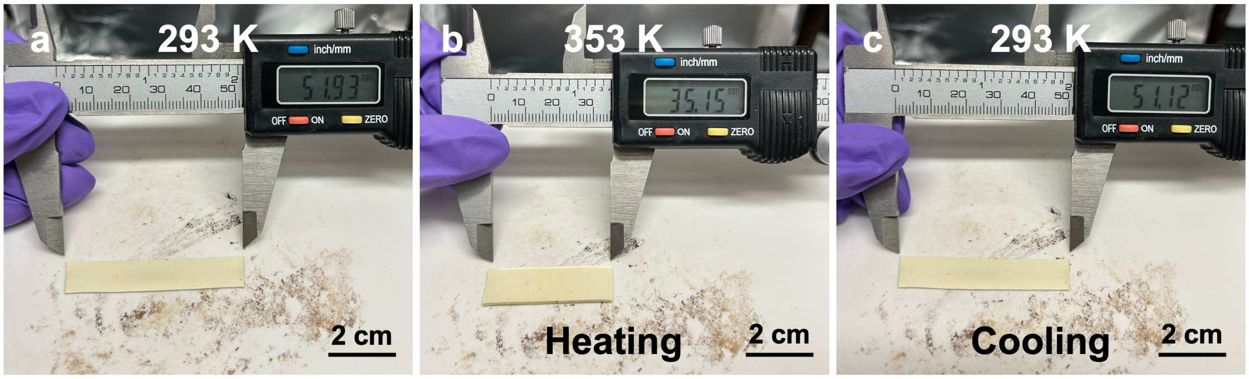

Fig. S2. Photos showing the reversible contraction and elongation of the monodomain LCE/PZT film (27.1 wt% PZT) upon heating and cooling. (a) At the room temperature, 293 K, the film length is 51.93 mm. (b) After heating to 353 K for 1 min, the film length is 35.15 mm. (c) After cooling back to 293 K, the film length is recovered to 51.12 mm.

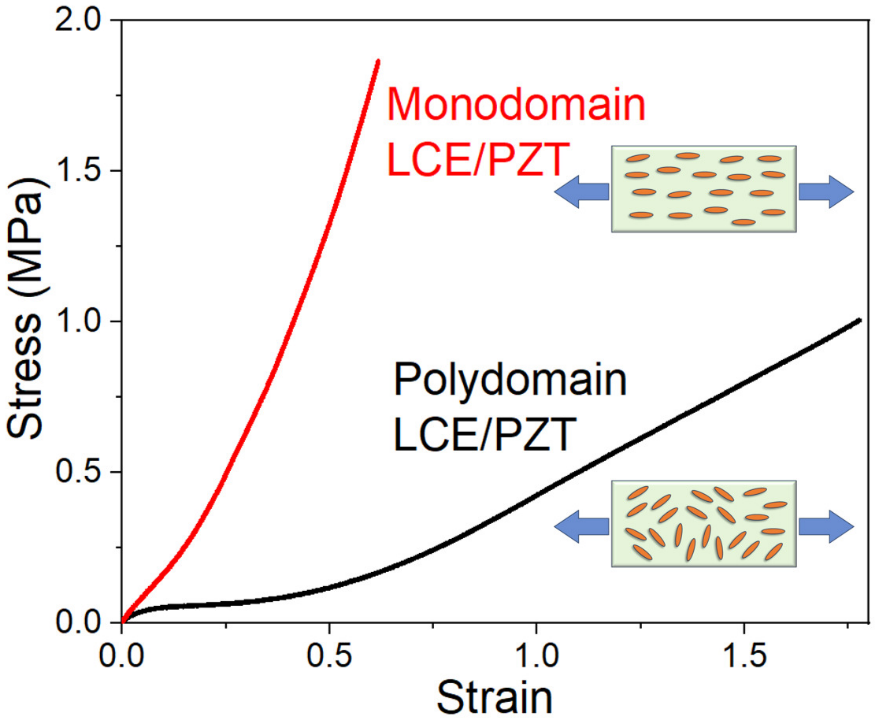

Fig. S3. Stress-strain curves in tensile tests of monodomain and polydomain LCE/PZT (27.1 wt% PZT). Inset: Schematics showing that the stress direction is the longitudinal direction of the LCE/PZT films, which is parallel to the alignment direction of the monodomain LCE/PZT.

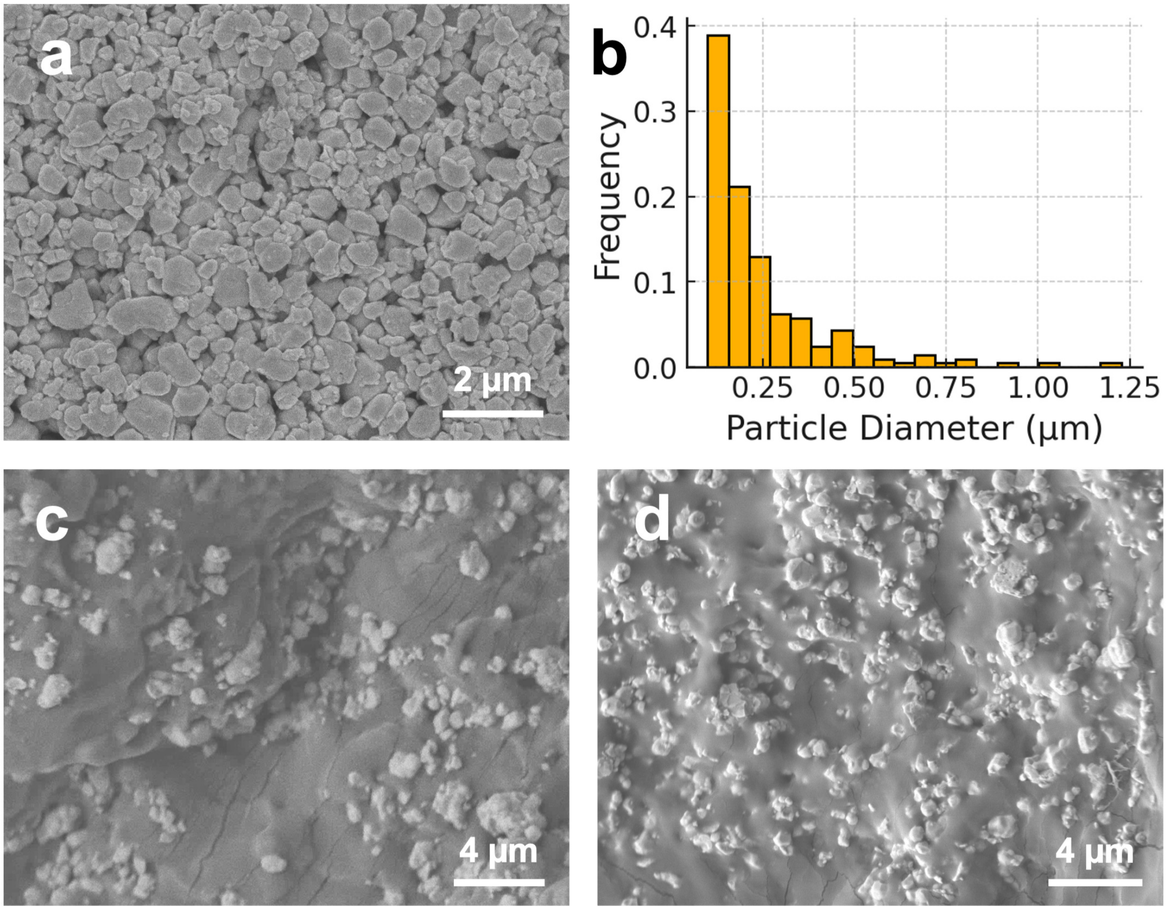

Fig. S4. Size distribution of PZT NPs after ball-milling and their dispersions in the LCE matrix. (a) SEM image of the PZT NPs after ball-milling. (b) A graph showing the size distribution of the PZT NPs in (a). Average diameter: 0.24 µm. Standard deviation: 0.17 µm. (c-d) Cross-sectional SEM images of the LCE/PZT films of different PZT NP loadings. (c) 15.7 wt%, and (d) 27.1 wt%.

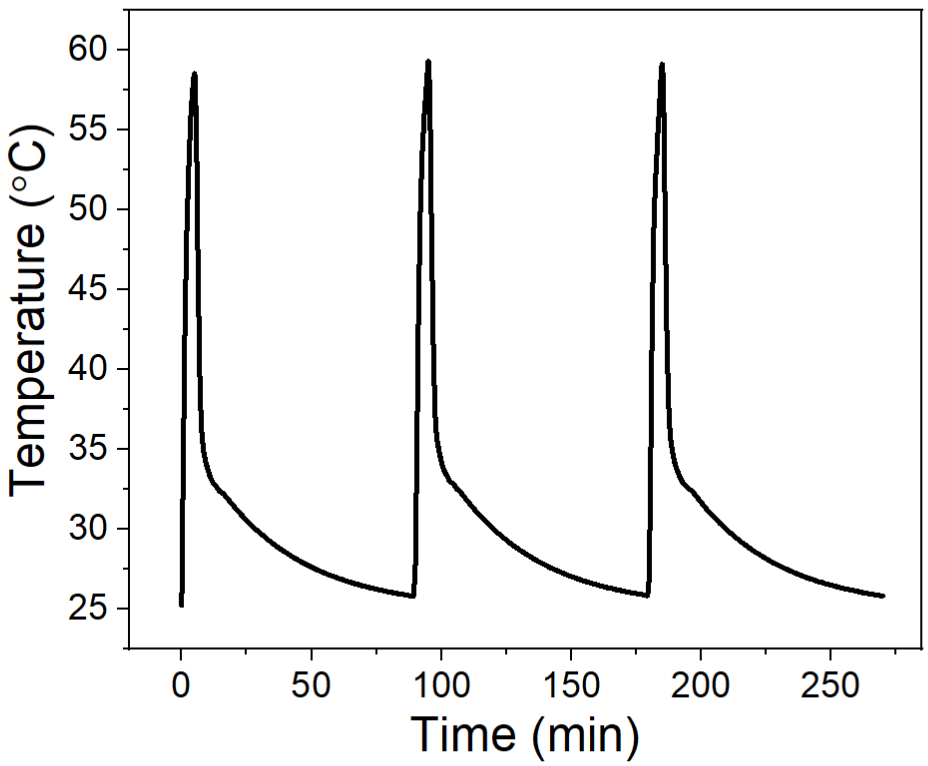

Fig. S5. The cyclic temperature profiles with temperatures varied between 25 °C to 58 °C. In each cycle, heating to 58 °C takes 5 min and cooling to 25 °C takes 85 min.

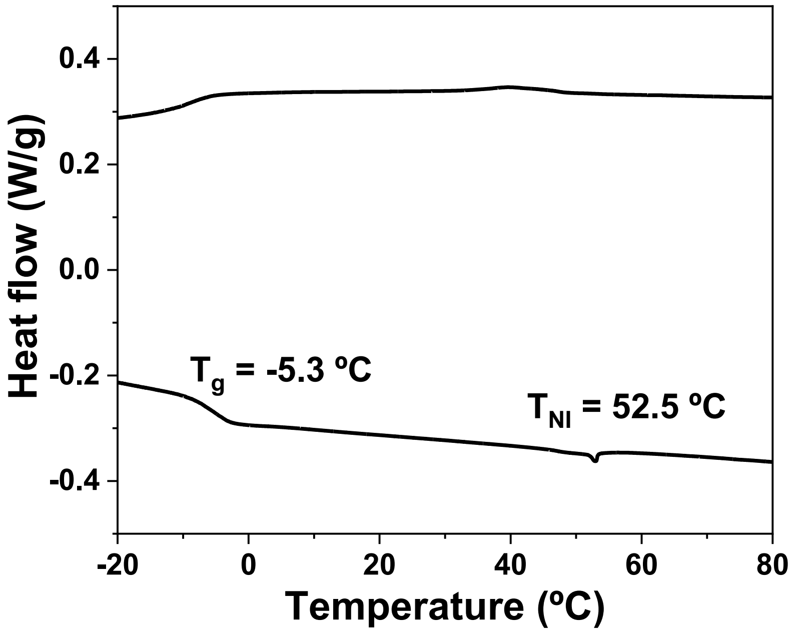

Fig. S6. Differential scanning calorimetry (DSC) curves the polydomain LCE sample. The glass transition temperature (T_g) and the nematic to isotropic phase transition temperature (T_{NI}) are -5.3 °C and 52.5 °C, respectively.

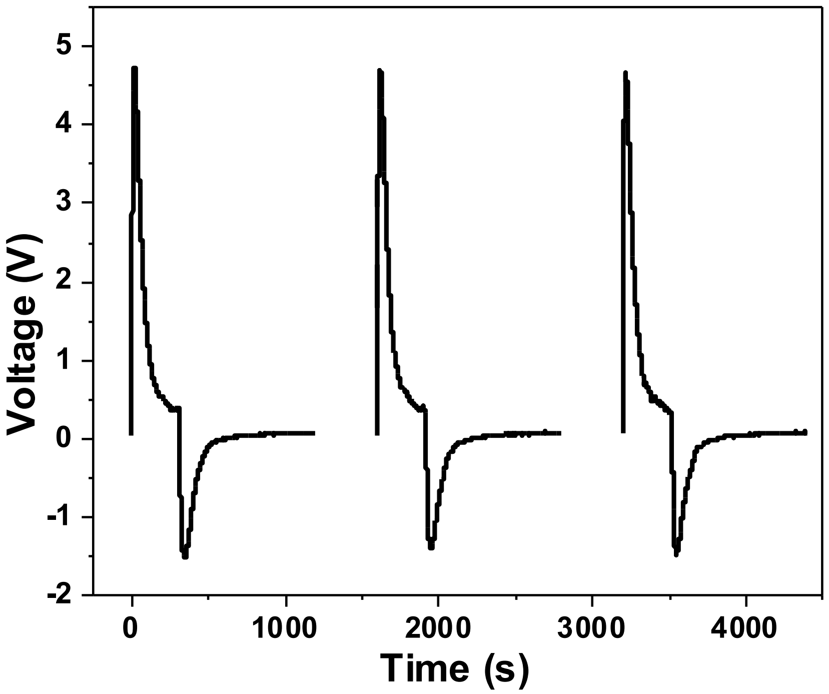

Fig. S7. The voltage profile of the polydomain LCE/PZT composite with 27.1 wt% PZT NPs, measured at 10 GΩ impedance.

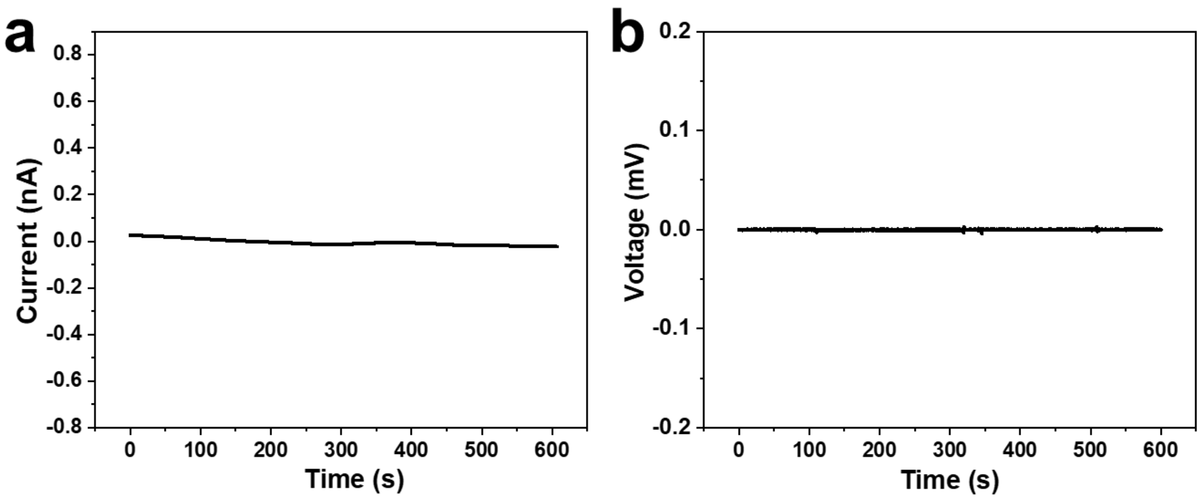

Fig. S8. Pyroelectric output of the pure polydomain LCE. (a) The short-circuit current profile and (b) the open-circuit voltage profile when subjected to the same heating and cooling cycles shown in Figure 2a.

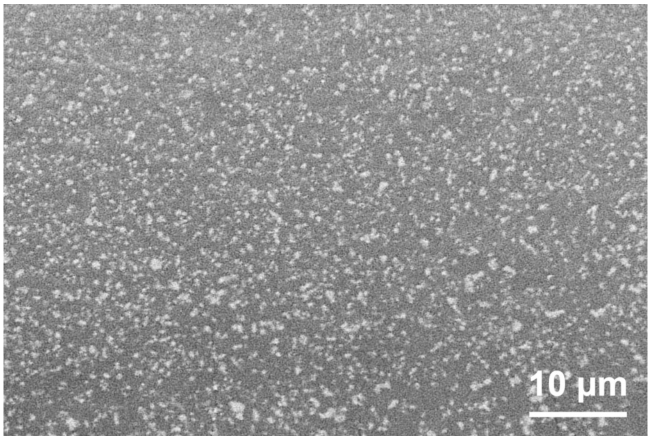

Fig. S9. Cross-sectional SEM image of the LCE/$BaTiO_3$ film with 27.1 wt% $BaTiO_3$ loading.

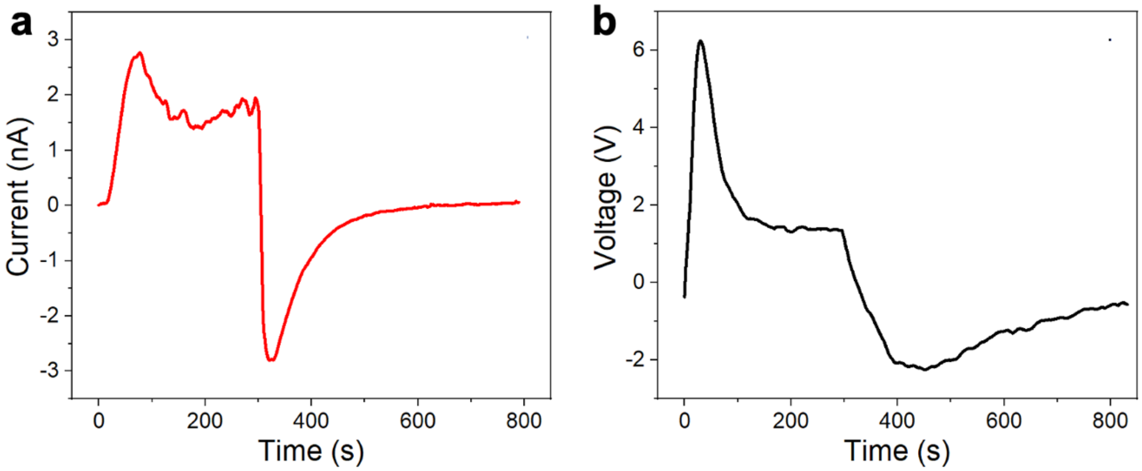

Fig. S10. The current and voltage profiles of the monodomain LCE/PZT composite (42.7 wt% PZT NPs) with fixed film boundary conditions. (a) Current and (b) voltage. The voltage is measured with a voltmeter at 10 GΩ impedance.

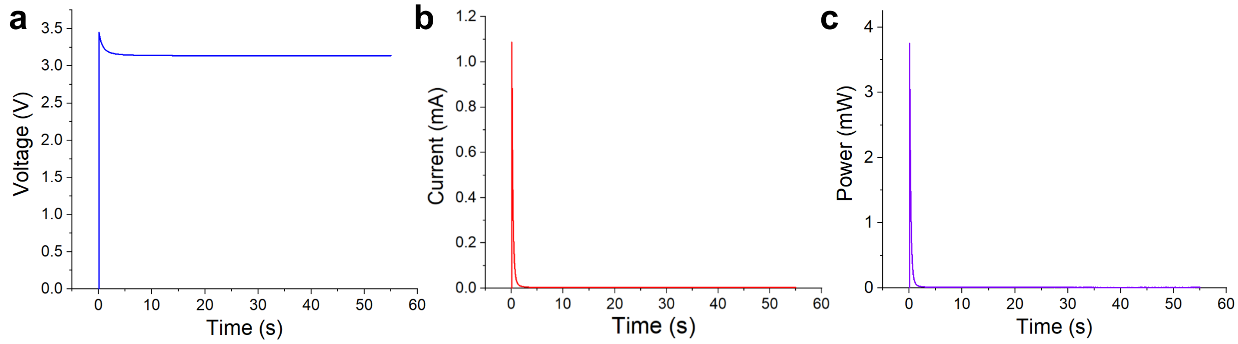

Fig. S11. The voltage, current, and power profiles of the green LED used in the demonstration shown in Figure 6. (a) Voltage, (b) current, and (c) power.

Supplementary Table

Table S1. A comparison of the pyroelectric coefficients of the state-of-the-art flexible pyroelectric materials with this work.

Flexible pyroelectric materials	Pyroelectric coefficient p ($nC \cdot cm^{-2} \cdot K^{-1}$)	References
LCE/PZT	-4.01	This work
PDMS/$KNbO_3$ nanowire	-0.800	(*10*)
P(VDF-TrFE)	~ -0.0450	(*14*)
PVDF	~ -1.40	(*15*)
PVDF	-2.70	(*42*)
PVDF	-3.00	(*43*)
PVDF	-3.00	(*44*)
PVDF nanofiber	~ -0.110	(*45*)
P(VDF-TrFE) nanowire	-3.19	(*46*)
Adiprene/PZT	-2.25	(*47*)
PVA/PZT	-1.10	(*48*)
PVA-PAA/PZT	-2.85	(*49*)

Table S2. The mechanical performance and pyroelectric current density of the monodomain LCE/PZT film (27.1 wt% PZT) at different LCE compositions.

Recipe	#1	#2	#3	#4	#5	#6
RM257 (g)	5.00	5.00	5.00	5.00	5.00	5.00
EDDT (g)	1.31	1.294	1.276	1.216	1.22	1.116
PETMP (g)	0.216	0.24	0.266	0.25	0.34	0.23
Toluene (g)	1.55	1.55	1.55	1.55	1.55	1.55
DPA, 2 wt% (g)	0.81	0.81	0.81	0.81	0.81	0.81
ACHN (g)	0.15	0.15	0.15	0.15	0.15	0.15
PZT (g)	2.50	2.50	2.50	2.50	2.50	2.50
Molar ratio of EDDT to PETMP	16.3	14.5	13	13	9.6	13
Molar ratio of acrylates to thiols	1.05	1.05	1.05	1.1	1.05	1.2
Young's modulus (MPa)	0.66	1.05	1.70	2.19	Brittle, fractured during the 2-step crosslinking	Liquid-like, unable to perform 2-step crosslinking
Thermal expansion coefficient (K^{-1})	-0.0059	-0.0054	-0.0050	-0.0042		
Simulated current density (nA/cm^2)	0.38	0.40	0.44	0.45		
Measured current density (nA/cm^2)	0.46	0.47	0.54	0.54		

Movie S1.
Lighting a green LED with the LCE/PZT thermal energy harvester for 9 continuous cycles.

REFERENCES AND NOTES

1. Lawrence Livermore National Laboratory, Estimated U.S. Energy Consumption in 2022: 100.3 Quads. https://flowcharts.llnl.gov/sites/flowcharts/files/2023-10/US%20Energy%202022.pdf [accessed October 2023].

2. X. L. Shi, J. Zou, Z. G. Chen, Advanced thermoelectric design: From materials and structures to devices. *Chem. Rev.* **120**, 7399–7515 (2020).

3. D. Zhang, H. Wu, C. R. Bowen, Y. Yang, Recent advances in pyroelectric materials and applications. *Small* **17**, e2103960 (2021).

4. H. Ryu, S. W. Kim, Emerging pyroelectric nanogenerators to convert thermal energy into electrical energy. *Small* **17**, e1903469 (2021).

5. S. B. Lang, Pyroelectricity: From ancient curiosity to modern imaging tool. *Phys. Today* **58**, 31–36 (2005).

6. G. Velarde, S. Pandya, L. Zhang, D. Garcia, E. Lupi, R. Gao, J. D. Wilbur, C. Dames, L. W. Martin, Quantifying intrinsic, extrinsic, dielectric, and secondary pyroelectric responses in $PbZr_{1-x}Ti_xO_3$ thin films. *ACS Appl. Mater. Interfaces* **11**, 35146–35154 (2019).

7. Y. Yang, W. Guo, K. C. Pradel, G. Zhu, Y. Zhou, Y. Zhang, Y. Hu, L. Lin, Z. L. Wang, Pyroelectric nanogenerators for harvesting thermoelectric energy. *Nano Lett.* **12**, 2833–2838 (2012).

8. N. Ma, Y. Yang, Enhanced self-powered UV photoresponse of ferroelectric $BaTiO_3$ materials by pyroelectric effect. *Nano Energy* **40**, 352–359 (2017).

9. Y. Yang, S. Wang, Y. Zhang, Z. L. Wang, Pyroelectric nanogenerators for driving wireless sensors. *Nano Lett.* **12**, 6408–6413 (2012).

10. Y. Yang, J. H. Jung, B. K. Yun, F. Zhang, K. C. Pradel, W. Guo, Z. L. Wang, Flexible pyroelectric nanogenerators using a composite structure of lead-free $KNbO_3$ nanowires. *Adv. Mater.* **24**, 5357–5362 (2012).

11. F. Gao, W. Li, X. Wang, X. Fang, M. Ma, A self-sustaining pyroelectric nanogenerator driven by water vapor. *Nano Energy* **22**, 19–26 (2016).

12. A. Cuadras, M. Gasulla, V. Ferrari, Thermal energy harvesting through pyroelectricity. *Sens. Actuators A Phys.* **158**, 132–139 (2010).

13. H. Xue, Q. Yang, D. Wang, W. Luo, W. Wang, M. Lin, D. Liang, Q. Luo, A wearable pyroelectric nanogenerator and self-powered breathing sensor. *Nano Energy* **38**, 147–154 (2017).

14. J. H. Lee, K. Y. Lee, M. K. Gupta, T. Y. Kim, D. Y. Lee, J. Oh, C. Ryu, W. J. Yoo, C. Y. Kang, S. J. Yoon, J. B. Yoo, S. W. Kim, Highly stretchable piezoelectric-pyroelectric hybrid nanogenerator. *Adv. Mater.* **26**, 765–769 (2014).

15. S. Wang, Z. L. Wang, Y. Yang, A one-structure-based hybridized nanogenerator for scavenging mechanical and thermal energies by triboelectric–piezoelectric–pyroelectric effects. *Adv. Mater.* **28**, 2881–2887 (2016).

16. I. M. McKinley, L. Pilon, Phase transitions and thermal expansion in pyroelectric energy conversion. *Appl. Phys. Lett.* **102**, 023906 (2013).

17. H. Li, C. R. Bowen, Y. Yang, Phase transition enhanced pyroelectric nanogenerators for self-powered temperature sensors. *Nano Energy* **102**, 107657, (2022).

18. Y. Wang, J. Liu, S. Yang, Multi-functional liquid crystal elastomer composites. *Appl. Phys. Rev.* **9**, 011301 (2022).

19. S. V. Fridrikh, E. M. Terentjev, Polydomain-monodomain transition in nematic elastomers. *Phys. Rev. E* **60**, 1847–1857 (1999).

20. Y. Wang, R. Yin, L. Jin, M. Liu, Y. Gao, J. Raney, S. Yang, 3D-printed photoresponsive liquid crystal elastomer composites for free-form actuation. *Adv. Funct. Mater.* **33**, 2210614 (2023).

21. L. Li, X. Dong, J. Xu, Y. Jiang, X. Zhou, Q. Li, N. Yuan, J. Ding, Liquid-phase drawing of LCE/CNT composites for electrothermal actuators. *Sens. Actuators B Chem.* **390**, 133846 (2023).

22. Y. Wu, S. Zhang, Y. Yang, Z. Li, Y. Wei, Y. Ji, Locally controllable magnetic soft actuators with reprogrammable contraction-derived motions. *Sci. Adv.* **8**, eabo6021 (2022).

23. J. M. Haberl, A. Sánchez-Ferrer, A. M. Mihut, H. Dietsch, A. M. Hirt, R. Mezzenga, Liquid-crystalline elastomer-nanoparticle hybrids with reversible switch of magnetic memory. *Adv. Mater.* **25**, 1787–1791 (2013).

24. A. Agrawal, H. Chen, H. Kim, B. Zhu, O. Adetiba, A. Miranda, A. C. Chipara, P. M. Ajayan, J. G. Jacot, R. Verduzco, Electromechanically responsive liquid crystal elastomer nanocomposites for active cell culture. *ACS Macro Lett.* **5**, 1386–1390 (2016).

25. M. Zadan, D. K. Patel, A. P. Sabelhaus, J. Liao, A. Wertz, L. Yao, C. Majidi, Liquid crystal elastomer with integrated soft thermoelectrics for shape memory actuation and energy harvesting. *Adv. Mater.* **34**, e2200857 (2022).

26. Z. Huang, L. Li, T. Wu, T. Xue, W. Sun, Q. Pan, H. Wang, H. Xie, J. Chi, T. Han, X. Hu, M. Su, Y. Chen, Y. Song, Wearable perovskite solar cells by aligned liquid crystal elastomers. *Nat. Commun.* **14**, 1204 (2023).

27. W. Wei, J. Gao, J. Yang, J. Wei, J. Guo, A NIR light-triggered pyroelectric-dominated generator based on a liquid crystal elastomer composite actuator for photoelectric conversion and self-powered sensing. *RSC Adv.* **8**, 40856–40865 (2018).

28. Y. Han, C. Jiang, H. Fu, C. Luo, H. Lin, H. Peng, A flexible bilayer actuator based on liquid crystal network and PVDF–TrFE for low-grade waste heat harvesting. *Energ. Technol.* **8**, 2000612 (2020).

29. J. Zhao, L. Zhang, J. Hu, Varied alignment methods and versatile actuations for liquid crystal elastomers: A review. *Adv. Intell. Syst.* **4**, 2100065 (2022).

30. G. L. Oliveira, C. A. Costa, S. C. S. Teixeira, M. F. Costa, The use of nano- and micro-instrumented indentation tests to evaluate viscoelastic behavior of poly(vinylidene fluoride) (PVDF). *Polym. Test.* **34**, 10–16 (2014).

31. B. Mohammadi, A. A. Yousefi, S. M. Bellah, Effect of tensile strain rate and elongation on crystalline structure and piezoelectric properties of PVDF thin films. *Polym. Test.* **26**, 42–50 (2007).

32. Y. Fu, E. C. Harvey, M. K. Ghantasala, G. M. Spinks, Design, fabrication and testing of piezoelectric polymer PVDF microactuators. *Smart Mater. Struct.* **15**, S141–S146 (2006).

33. Y. Wang, A. Dang, Z. Zhang, R. Yin, Y. Gao, L. Feng, S. Yang, Repeatable and reprogrammable shape morphing from photoresponsive gold nanorod/liquid crystal elastomers. *Adv. Mater.* **32**, e2004270 (2020).

34. M. Liu, L. Jin, S. Yang, Y. Wang, C. B. Murray, S. Yang, Shape morphing directed by spatially encoded, dually responsive liquid crystalline elastomer micro-actuators. *Adv. Mater.* **35**, e2208613 (2023).

35. J. Liu, Y. Gao, H. Wang, R. Poling-Skutvik, C. O. Osuji, S. Yang, Shaping and locomotion of soft robots using filament actuators made from liquid crystal elastomer–carbon nanotube composites. *Adv. Intell. Syst.* **2**, 1900163 (2020).

36. H. Cui, R. Hensleigh, D. Yao, D. Maurya, P. Kumar, M. G. Kang, S. Priya, X. Zheng, Three-dimensional printing of piezoelectric materials with designed anisotropy and directional response. *Nat. Mater.* **18**, 234–241 (2019).

37. K. Kim, J. L. Middlebrook, J. E. Chen, W. Zhu, S. Chen, D. J. Sirbuly, Tunable surface and matrix chemistries in optically printed (0-3) piezoelectric nanocomposites. *ACS Appl. Mater. Interfaces* **8**, 33394–33398 (2016).

38. C. R. Bowen, J. Taylor, E. LeBoulbar, D. Zabek, A. Chauhanc, R. Vaish, Pyroelectric materials and devices for energy harvesting applications. *Energ. Environ. Sci.* **7**, 3836–3856 (2014).

39. Q. Li, S. Li, D. Pisignano, L. Persano, Y. Yang, Y. Su, On the evaluation of output voltages for quantifying the performance of pyroelectric energy harvesters. *Nano Energy* **86**, 106045 (2021).

40. S. Gupta, R. Bhunia, B. Fatma, D. Maurya, D. Singh, R. Gupta, S. Priya, R. K. Gupta, A. Garg, Multifunctional and flexible polymeric nanocomposite films with improved ferroelectric and piezoelectric properties for energy generation devices. *ACS Appl. Energy Mater.* **2**, 6364–6374 (2019).

41. H. Yan, Y. He, L. Yao, X. Wang, X. Zhang, Y. Zhang, D. Han, C. Li, L. Sun, J. Zhang, Thermo-crosslinking assisted preparation of thiol-acrylate main-chain liquid-crystalline elastomers. *J. Polym. Res.* **29**, 450 (2022).

42. R.W. Whatmore, Pyroelectric devices and materials. *Rep. Prog. Phys.* **49**, 1335–1386 (1986).

43. H. Zhang, Y. Xie, X. Li, Z. Huang, S. Zhang, Y. Su, B. Wu, L. He, W. Yang, Y. Lin. Flexible pyroelectric generators for scavenging ambient thermal energy and as self-powered thermosensors. *Energy* **101**, 202–210 (2016).

44. T. Zhao, W. Jiang, D. Niu, H. Liu, B. Chen, Y. Shi, L. Yin, B. Lu. Flexible pyroelectric device for scavenging thermal energy from chemical process and as self-powered temperature monitor. *Appl. Energy* **195**, 754–760 (2017).

45. M. H. You, X. X. Wang, X. Yan, J. Zhang, W. Z. Song, M. Yu, Z. Y. Fan, S. Ramakrishna, Y. Z. Long, A self-powered flexible hybrid piezoelectric–pyroelectric nanogenerator based on non-woven nanofiber membranes. *J. Mater. Chem. A* **6**, 3500–3509 (2018).

46. X. Chen, J. Shao, X. Li, H. Tian, A flexible piezoelectric-pyroelectric hybrid nanogenerator based on P(VDF-TrFE) nanowire array. *IEEE Trans. Nanotechnol.* **15**, 295–302 (2016).

47. A. S. Bhalla, R. E. Newnham, L. E. Cross, W. A. Schulze, J. P. Dougherty, W. A. Smith, Pyroelectric PZT-polymer composites. *Ferroelectrics* **33**, 139–146 (1981).

48. S. Uma, J. Philip, Induction of electro-activity in polyvinyl alcohol with addition of nanocrystalline PZT ceramic. *Ind. J. Pure Appl. Phys.* **51**, 717–723 (2013).

49. S. Uma, J. Philip, Enhancement in pyroelectric properties of PZT-PVA polymer nanocomposites with addition of PAA. *J. Appl. Polym. Sci.* **131**, 41142 (2014).
